# Direct regeneration of degraded lithium-ion battery cathodes with a multifunctional organic lithium salt

**DOI:** 10.1038/s41467-023-36197-6

**Published:** 2023-02-03

**Authors:** Guanjun Ji, Junxiong Wang, Zheng Liang, Kai Jia, Jun Ma, Zhaofeng Zhuang, Guangmin Zhou, Hui-Ming Cheng

**Affiliations:** 1 Tsinghua-Berkeley Shenzhen Institute & Tsinghua Shenzhen International Graduate School, Tsinghua University, Shenzhen, 518055 China; 2 Frontiers Science Center for Transformative Molecules, School of Chemistry and Chemical Engineering, Shanghai Jiao Tong University, Shanghai, 200240 China; 3 Faculty of Materials Science and Engineering/Institute of Technology for Carbon Neutrality, Shenzhen Institute of Advanced Technology, Chinese Academy of Science, Shenzhen, 518055 China; 4 Shenyang National Laboratory for Materials Science, Institute of Metal Research, Chinese Academy of Sciences, Shenyang, 110016 China

**Keywords:** Batteries, Batteries, Batteries

## Abstract

The recycling of spent lithium-ion batteries is an effective approach to alleviating environmental concerns and promoting resource conservation. LiFePO_4_ batteries have been widely used in electric vehicles and energy storage stations. Currently, lithium loss, resulting in formation of Fe(III) phase, is mainly responsible for the capacity fade of LiFePO_4_ cathode. Another factor is poor electrical conductivity that limits its rate capability. Here, we report the use of a multifunctional organic lithium salt (3,4-dihydroxybenzonitrile dilithium) to restore spent LiFePO_4_ cathode by direct regeneration. The degraded LiFePO_4_ particles are well coupled with the functional groups of the organic lithium salt, so that lithium fills vacancies and cyano groups create a reductive atmosphere to inhibit Fe(III) phase. At the same time, pyrolysis of the salt produces an amorphous conductive carbon layer that coats the LiFePO_4_ particles, which improves Li-ion and electron transfer kinetics. The restored LiFePO_4_ cathode shows good cycling stability and rate performance (a high capacity retention of 88% after 400 cycles at 5 C). This lithium salt can also be used to recover degraded transition metal oxide-based cathodes. A techno-economic analysis suggests that this strategy has higher environmental and economic benefits, compared with the traditional recycling methods.

## Abstract (web summary)

Sustainable recycle of spent Li ion batteries is an effective strategy to alleviate environmental concerns and support resource conservation. Here, authors report the direct regeneration of LiFePO_4_ cathode using multifunctional organic lithium salts, leading to high environmental and economic benefits.

## Introduction

The pursuit of higher energy density and better safety has been the dominant focus for lithium-ion battery (LIB) manufacturers in recent years^1–5^. Lithium iron phosphate (LiFePO_4_, LFP) batteries have attracted attention due to their structural stability, long service life, and emerging cell-to-pack technological breakthroughs^6^. These advantages enable LFP batteries to be widely used in electric vehicles and energy storage stations, while also leading to an increased number of spent LIBs^7–9^. The current end-of-life battery recycling technologies include pyrometallurgical recycling (pyro-), hydrometallurgical recycling (hydro-), and direct regeneration (direct)^10–12^. The pyro-route generally requires high-temperature smelting and a lot of energy to produce a metal alloy, while the hydro-route consumes strong acid/alkali reagents and needs many steps to obtain a high-purity precursor^13–15^. Specifically, the only valuable element in the degraded LFP battery is lithium, and the low economic value of the other compounds produced in current recycling methods means that better technologies are needed. In addition, the energy consumption and greenhouse gas emissions involved must be taken into consideration^16–18^.

**Fig. 1:**
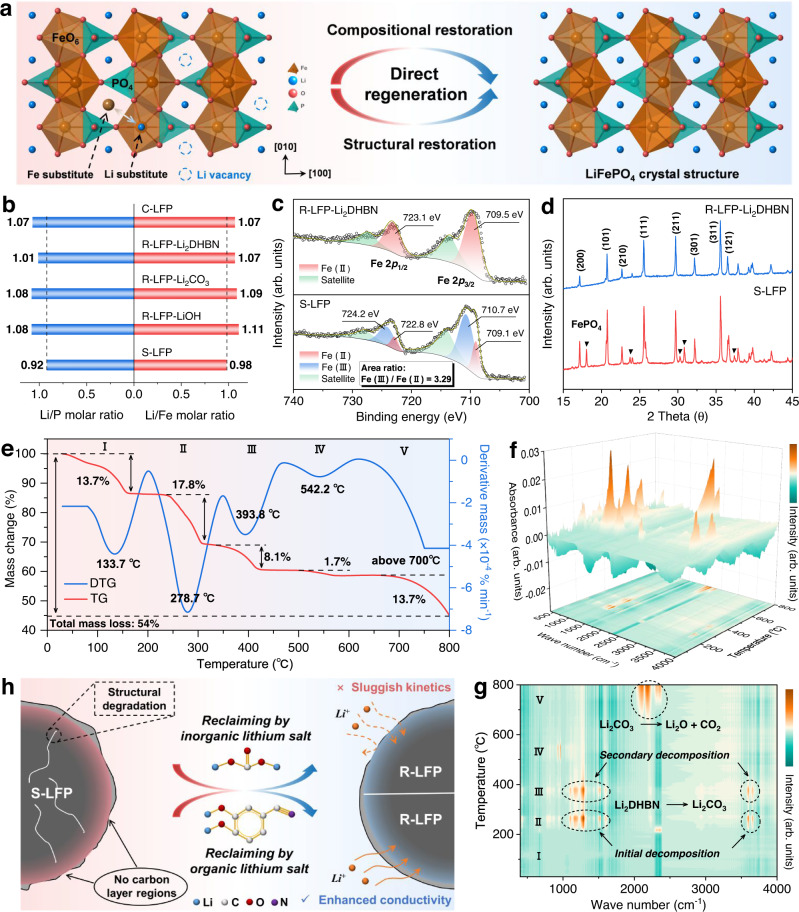
Degradation mechanism and regeneration process of LFP cathodes. **a** Schematic of the degraded and restored crystal structures. **b** Li/P and Li/Fe molar ratio based on ICP-OES. **c** Fe 2*p* XPS spectrum of S-LFP and R-LFP-Li_2_DHBN. **d** XRD spectra of S-LFP and R-LFP-Li_2_DHBN. **e** TG-DTA, **f** 3D IR map, **g** IR contour plot of Li_2_DHBN based on TG-IR coupling measurements. **h** Schematic of the regeneration mechanism of S-LFP by using inorganic and organic lithium salts.

Direct regeneration is an effective strategy aimed at restoring the composition and structure of the degraded cathode material to its original state, which achieves the maximum sustainability of spent LIBs. Understanding the failure mechanism is crucial to restoring LFP cathode materials. Generally, the loss of lithium is the dominant reason, resulting in a capacity decay after long-term cycling^19,20^. Lithium loss causes the formation of Fe(III) in the bulk crystal structure, thus inducing some Li-Fe anti-site defects (Fig. 1a). Previous studies have found that direct recycling of the degraded LFP is achieved by targeted healing^19^, using a prelithiated separator^21^, and by a molten salt process^22^. During the regeneration, compensating for the lost lithium and constructing a reduction environment are considered two critical factors^22–25^. Moreover, sluggish charge transport is an inherent disadvantage of LFP cathode materials, which is directly related to the rate performance^26^. Coating with electronically conductive agents like carbon and conducting polymers is a widely used modification strategy^8,27^. However, the carbon coating on the particle surface will be destroyed after long-term cycles, leading to a partially deficient and noncontinuous conducting network in the degraded LFP particles.

Addressing the above three problems is key to restoring the composition and structure of the degraded LFP cathode in direct regeneration. Although inorganic lithium salts (Li_2_CO_3_^28,29^, LiOH^19,24,30^, Li_2_SO_4_^23^) have been commonly used as a lithium source to compensate for the loss, the monofunctional characteristic limits them from serving as the reducing agent or carbon source. Currently, extra carbon needs to be added to improve the poor electrical conductivity of LFP in the regeneration process, which is not economical^27,31,32^. Hence, it is critical to look for a lithium salt that not only compensates for the lost Li, but also plays an important role in structural restoration. First, lithium must be able to separate from the host and easily fill the vacancy. Second, the lithium salt itself should be reductive or have functional groups with strong reductive properties to decrease the amount of Fe(III) in the bulk and surface structures. More importantly, the lithium salt had better enable the recycled materials re-coat the conductive network to improve the ion/electron transfer.

In this work, we use a multifunctional organic lithium salt (3,4-dihydroxybenzonitrile dilithium, Li_2_DHBN) to restore the degraded LFP cathode materials by a direct regeneration process. This salt couples well with the surface of the damaged LFP particles by functional groups at a low temperature. An increased temperature enables Li-ions to enter the vacancies, and cyano groups in the Li_2_DHBN create a reductive atmosphere to decrease Fe(III) formation. Meanwhile, the amorphous carbon forms during the pyrolysis of the salt at a high temperature and coats on the surface of the damaged LFP particles. The composition and structure of the LFP are simultaneously restored in one step, and the introduction of other reductive or carbon sources is not required. The recycled LFP cathode has a superior rate performance and kinetics to that achieved by another lithium salt. This organic lithium salt can also be used to restore highly degraded transition metal oxide-based cathodes (LiCoO_2_ and LiNi_0.5_Co_0.2_Mn_0.3_O_2_). Our proposed strategy is cost-effective and competitive compared with traditional recycling methods and opens up opportunities for future spent LIB recycling.

## Results

### Degradation mechanism and regeneration process of LiFePO_4_ cathodes

The degraded LIBs used in the recovery process and the corresponding basic parameters are listed in Supplementary Table 1, and the detailed disassembly procedure is shown in Supplementary Fig. 1. The composition and phase of S-LFP were first analyzed to determine the degree of failure and the internal mechanism. Inductively coupled plasma-optical emission spectrometry (ICP-OES) results (Fig. 1b and Supplementary Table 2) show an obvious lithium deficiency in S-LFP compared with the regenerated LFP cathodes (R-LFP) and a commercial LFP cathode. The loss of lithium not only causes the capacity fade but also induces partial Fe(III) formation. Fe 2*p* X-ray photoelectron spectroscopy (XPS) (Fig. 1c) was used to determine the valence change of Fe on the surface of LFP. For S-LFP, the main peaks at 710.7 eV and 709.1 eV are respectively attributed to the Fe(III) and Fe(II) of Fe 2*p*_3/2_. The presence of Fe(II) is due to the inhomogeneity of phase distributions in S-LFP surface after long-term cycles^19,33^. Quantification of Fe(III)/Fe(II) was presented by the Fe 2*p*_3/2_ peak fitting area ratio, suggesting that the main phase is FePO_4_ on the surface of degraded LFP particles. An obvious FePO_4_ phase was also observed in X-ray diffraction (XRD) results (Fig. 1d and Supplementary Fig. 2). For R-LFP-Li_2_DHBN, the main peaks of Fe 2*p*_3/2_ and Fe 2*p*_1/2_ shift to lower binding energy compared with S-LFP. The fitting peak at 709.5 eV is ascribed to Fe(II) of Fe 2*p*_3/2_ and Fe(III) peak is not observed, indicating the compositional and structural restoration of R-LFP. In addition, Li/Fe anti-sites produce restricted diffusion paths for the lithium ions, thus limiting the reaction kinetics and rate properties^20,34^. An XPS survey (Supplementary Fig. 3) and scanning electron microscopy images (Supplementary Fig. 4) of S-LFP indicate that the active materials are mixed with conductive carbon and PVDF binder.

Thermogravimetry coupled with Fourier transform infrared (TG-FTIR) measurement was conducted to understand the decomposition process of the organic lithium salt. As shown by the TG result (Fig. 1e), decomposition occurs in five stages. Stage I at about 133.7 °C is ascribed to the loss of absorbed water. The following weight changes (stages II–IV) are mainly attributed to the decomposition of Li_2_DHBN and the formation of Li_2_CO_3_, corresponding to the FTIR contour map (Fig. 1f, g). Other compounds containing carbon and nitrogen are also forming, which is proved by the XRD result (Supplementary Fig. 6). Phase composition was determined to be carbon, Li_2_CO_3_, and a small amount of Li_2_O. Finally, the formed Li_2_CO_3_ decomposes to Li_2_O above 700 °C and participates in the following lithium supplementation process (Stage V). To simulate our experimental process, a mixture of S-LFP powder and Li_2_DHBN was also used in a TG-FTIR measurement (Supplementary Fig. 7) and the results agree with the above discussion. For comparison, traditional inorganic lithium salts were also used as the lithium source to restore the degraded LFP cathode but were ineffective (Noted R-LFP-Li_2_CO_3_, R-LFP-LiOH). The Li_2_DHBN not only restores the lithium vacancies and heals the bulk structure, but also forms a uniform carbon coating on the LFP (Fig. 1h) which improves the Li-ion and electron transfer kinetics, enabling the recycled LFP cathode to have a better electrochemical performance. It is this multifunctionality that makes our regeneration process superior.

**Fig. 2:**
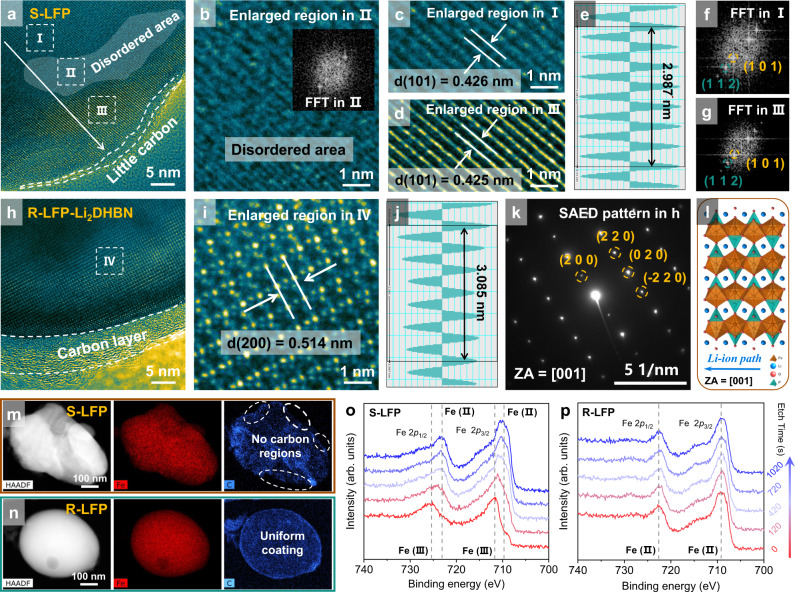
Microstructure characterization of the degraded and restored LFP cathodes. **a** HRTEM image, **b**–**d** enlarged figures, **e** line profiles in **d** and **f**, **g** fast Fourier transform (FFT) images of S-LFP. **h** HRTEM, **i** enlarged figure, **j** line profiles in **i** and **k** SAED pattern of R-LFP-Li_2_DHBN. **l** Schematic of the LFP crystal structure viewed along [001]. **m**, **n** HAADF-STEM images and EDS maps of S-LFP and R-LFP-Li_2_DHBN. In-depth Fe 2*p* XPS spectrum of **o** S-LFP and **p** R-LFP-Li_2_DHBN.

High-resolution transmission electron microscopy (HRTEM) images show the microstructure of LFP particles at the atomic level. For S-LFP, an enlarged view of the edge shows the places where there is no carbon coating (Fig. 2a and Supplementary Fig. 8a, b) probably due to electrolyte erosion during the long-term cycling. Mechanical interaction may also result in irrecoverable damage to the surface in some recycling procedures. These phenomena may either destroy the carbon layer or cause it to separate from the LFP causing a deterioration of the reaction interface. Figure 2b shows that disordered areas gradually appear on the surface of the LFP particles, suggesting their structural degradation. Enlarged images in Fig. 2c, d and FFT images in Fig. 2e–g show a lattice spacing of 0.425 nm which corresponds to the (101) plane. For R-LFP-Li_2_CO_3_ there are still carbon loss regions since there is no source of extra carbon during the regeneration process (Supplementary Fig. 8c, d). Due to the high-temperature recrystallization and added lithium, the regenerated particle shows distinct lattice fringes (Supplementary Fig. 9) with a spacing of 0.288 nm, which is ascribed to the (301) plane of LFP.

For R-LFP-Li_2_DHBN, very regular particles coated by a carbon layer with a uniform thickness of 4–5 nm were seen in Fig. 2h and Supplementary Fig. 8e, f, where the carbon was derived from the pyrolysis of the organic lithium salt during heat treatment. This result was confirmed by energy dispersive spectroscopy (EDS) elemental mapping (Fig. 2m, n). In contrast, elemental carbon is not evenly distributed in S-LFP and R-LFP-Li_2_CO_3_ samples, especially on the particle surfaces (Supplementary Fig. 10). The lattice arrangement is consistent throughout the whole particle and the lattice spacing of 0.514 nm corresponds to the (200) crystal plane of LFP (Fig. 2i, j). Moreover, a selected area electron diffraction pattern taken along [001] shows obvious diffraction spots (Fig. 2k), corresponding to the LFP crystal structure in Fig. 2l. The EDS energy spectrum (Supplementary Fig. 11) and Fe 2*p* XPS (Fig. 1c) show that the phase composition is recovered to the original state and Fe(III) in the degraded materials is also eliminated after direct regeneration. In-depth XPS was further used to investigate the valence state of Fe from the surface to the bulk. For S-LFP, Fe 2*p*_3/2_ and Fe 2*p*_1/2_ peaks are respectively located at about 711.6 eV and 725.4 eV, ascribed to the Fe(III) phase (Fig. 2o). The main peaks shift to a lower binding energy with the increased etching time, suggesting Fe(III) phase in the surface of S-LFP. For R-LFP-Li_2_DHBN, the peaks at 709.2 eV and 722.6 eV, attributed respectively to Fe 2*p*_3/2_ and Fe 2*p*_1/2_ of Fe(II), remain unchanged from the surface to the bulk, indicating that FePO_4_ phase was completely eliminated after direct regeneration (Fig. 2p). It is, therefore, a clear advantage that the organic lithium salt not only restores the composition and structure, but also plays a significant role in recoating the conductive carbon layer.

### Electrochemical performance and kinetics of LiFePO_4_ cathodes

**Fig. 3:**
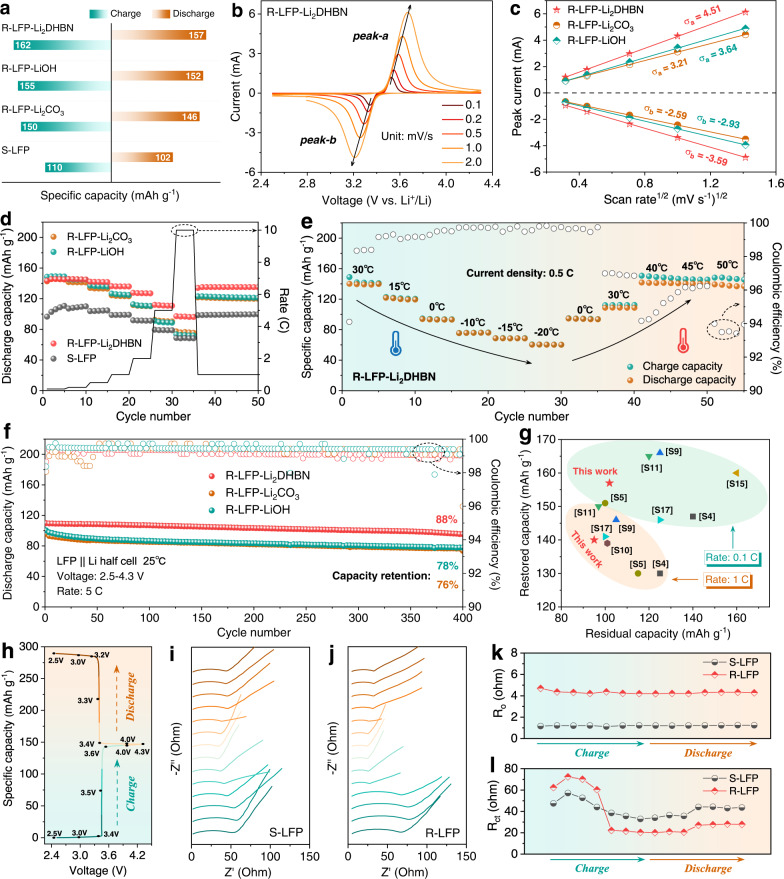
Electrochemical performance and kinetics of LFP cathodes. **a** The residual and restored capacity at 0.1 C. **b** CV curves of R-LFP-Li_2_DHBN at different scan rates. **c** Linear relationship between the major peak currents and scan rates. **d** Rate performance of S-LFP and R-LFP. **e** High-and-low temperature performance of R-LFP-Li_2_DHBN. **f** Long cycling performance at 5 C. **g** Comparison of residual capacity and restored capacity of LFP cathodes in this work and other published studies. **h** Voltage profile and **i**, **j** impedance spectra collected during the first cycle. **k**, **l** *R*_o_ and *R*_ct_ values from the fitted results of S-LFP and R-LFP-Li_2_DHBN.

The degraded sample had a discharge-specific capacity of 102 mAh g^−1^ at a current density of 0.1 C (1 C = 170 mA g^−1^), while those of the samples regenerated with R-LFP-Li_2_CO_3_, R-LFP-LiOH, and R-LFP-Li_2_DHBN were respectively 146, 152, 157 mAh g^−1^ (Fig. 3a). The S-LFP sample had a very poor cycling reversibility (Supplementary Fig. 12). The R-LFP-Li_2_DHBN sample has the smallest polarization voltage difference (206 mV) in the initial cyclic voltammetry (CV) curves, compared with R-LFP-Li_2_CO_3_ (218 mV) and R-LFP-LiOH (316 mV). The CV curves at different scan rates (Fig. 3b and Supplementary Fig. 13) show that the major peaks in charge and discharge are respectively due to the Li-ion de-insertion and insertion reactions. According to the Randles-Sevcik equation^35–37^,

1 $${I}_{p}=(2.65\times {10}^{5}){n}^{3/2}{{{SD}}_{{Li}}}^{1/2}{C}_{{Li}}{v}^{1/2}$$

where *I*_*p*_, *n*, *S*, *D*_*Li*_, *C*_*Li*_, and *v* are the peak current, number of electrons, area of the electrode, Li-ion diffusion coefficient, Li-ion concentration in the electrode, and voltage sweep rate, respectively. Specifically, *D*_*Li*_ is positively related to the slopes of the *I*_*p*_*/v*^*1/2*^ profiles. As shown in Fig. 3c, the slope for R-LFP-Li_2_DHBN is higher than for the other samples, suggesting a faster Li-ion diffusion rate. Furthermore, a galvanostatic intermittent titration technique test was used to calculate the *D*_*Li*_ value (Supplementary Fig. 14). The lithium-ion diffusion rate for R-LFP-Li_2_DHBN is the highest of all the regenerated samples during charge and discharge (Supplementary Fig. 15), indicating that the diffusion path blocks caused by Li/Fe anti-sites have been eliminated. Experiments showed that the optimum amount of organic lithium salt used and the reaction temperature are respectively 5 wt% and 800 °C (Supplementary Figs. 16,17). More of the salt provides adequate Li and a thicker layer of conductive carbon, but residual lithium salt remains on the surface of recycled materials and blocks the Li-ion diffusion paths, resulting in sluggish reaction kinetics.

The rate capability was also restored after direct regeneration (Fig. 3d). S-LFP had capacities of 98, 91, 79, and 68 mAh g^−1^ at current densities of 1, 2, 5, and 10 C, respectively. Although the discharge capacities of samples regenerated by inorganic lithium salts returned to the original levels for low current densities, the high-rate performance was still sluggish because of the loss of the conductive carbon. By contrast, R-LFP-Li_2_DHBN had a superior high-rate performance and capacities of 127, 111, and 97 mAh g^−1^ at 2, 5, and 10 C, respectively. Furthermore, high-and-low temperature tests (Fig. 3e) showed that R-LFP-Li_2_DHBN had a capacity of 61 mAh g^−1^ even at −20 °C and maintained good cycle stability at temperatures above 40 °C. This superior capability is attributed to the improved charge transfer produced by the carbon coating. Previous studies have found that coating the LFP particles with an electronically conductive agent improves the low-temperature properties of the electrode^27,31,32^. The long-term cycling performance was measured at 1 C rate (Supplementary Fig. 18). All samples had a similar performance after 300 cycles with a capacity retention of 90%, but when the current density reached 5 C, R-LFP-Li_2_DHBN still had a capacity of 110 mAh g^−1^ with a high retention of 88% after 400 cycles (Fig. 3f). In comparison, R-LFP-Li_2_CO_3_ and R-LFP-LiOH had respective capacities of only 73 and 77 mAh g^−1^ under the same conditions. Even at an ultrahigh 10 C rate, R-LFP-Li_2_DHBN kept stable during cycling, much better than that of the other recycled materials (Supplementary Fig. 19). Summary of direct regeneration methods and their performance of LFP cathodes is listed in Supplementary Table 3. In contrast, the LFP cathode used in our recovery process is highly degraded with a residual capacity of only 98 mAh g^−1^ at 1 C, which is the lowest of all published reports. The restored capacity and rate performance of R-LFP-Li_2_DHBN are competitive (Fig. 3g). Additionally, the Li_2_DHBN was also used to restore different types of spent LIB cathodes with different degrees of failure, such as LiCoO_2_ and LiNi_0.5_Co_0.2_Mn_0.3_O_2_ (Supplementary Fig. 20), thus providing a competitive lithium source for future direct regeneration technologies.

In-situ EIS measurements were carried out to clarify the change of internal resistance during the first cycle. Different voltages were collected during charge and discharge (Fig. 3h). Notably, the semicircles at a high frequency are ascribed to the charge transfer contribution from the cathode interfaces^38,39^. During charging, the charge transfer impedance (*R*_ct_) decreased gradually and reached a minimum at 100% state of charge (4.3 V). This resistance change reflects the increase of electronic and ionic conductivity of the LFP cathode during delithiation^40,41^. The change reversed when discharging. The cell resistance increased slightly and then remained at a steady state. The same process was observed for S-LFP and R-LFP, as shown in Fig. 3i, j and Supplementary Fig. 21. The fitted results of the equivalent circuit model for impedance parameters are listed in Supplementary Tables 4, 5. The inherent impedance of a coin cell (*R*_o_), which is associated with the assembly technology and other external factors, remains consistent during cycling (Fig. 3k). The *R*_ct_ value of R-LFP-Li_2_DHBN was much smaller than that of S-LFP during discharge (Fig. 3l), indicating that the reaction interface is more stable after direct regeneration.

### Analysis of the regeneration mechanism of LiFePO_4_ cathode using Li_2_DHBN

**Fig. 4:**
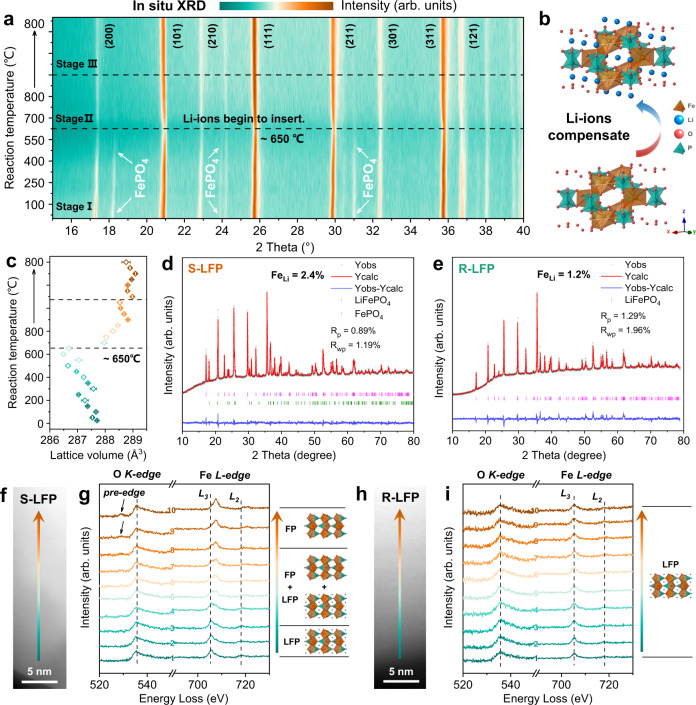
Analysis of the regeneration mechanism of LFP cathodes using Li_2_DHBN. **a** Contour plots of XRD patterns as a function of temperature in the selected 2 theta range. **b** Schematic showing that Li vacancies have been filled and crystalline structure recovers to the original state after direct regeneration. **c** Lattice volume changes from the in situ XRD results. **d**, **e** High-resolution powder XRD and Rietveld refinement results of S-LFP and R-LFP-Li_2_DHBN. **f**, **h** STEM images of S-LFP and R-LFP-Li_2_DHBN. O K-edge and Fe L-edge EELS spectra of **g** S-LFP, and **i** R-LFP-Li_2_DHBN.

In situ XRD measurements at different temperatures were used to monitor the change in phase composition (Fig. 4a), which is divided into three stages: (1) The organic lithium salt is gradually decomposed into lithium carbonate and other hydrocarbons in this temperature range, which has been confirmed by the above TG-IR results. Peaks for the FePO_4_ phase at 18.1° and 30.9° (2*θ*) gradually disappeared, demonstrating that the strongly-reducing cyano groups decrease the amount of Fe(III) (*Stage I*). (2) Lithium addition and structural restoration mainly occur in this stage. Lithium carbonate decomposes to lithium oxide and then lithium ions diffuse into the Li vacancies (Fig. 4b), associated with a sharp shift of the (111), (211), and (311) peaks to lower angles. The increased lattice volume is due to the lithium insertion (*Stage II*). (3) The LFP crystal structure gradually stabilizes and no more peak shifts are observed after direct regeneration (*Stage III*). The lattice volume changes in Fig. 4c also show that the LFP material has recovered to its original state. High-resolution XRD patterns show that S-LFP has a highly ordered *Pnma* space group with the coexistence of FePO_4_ and LiFePO_4_ phases (Fig. 4d). The detailed structural information in Supplementary Table 6 shows that the weight percentages of the FePO_4_ and LiFePO_4_ phases are respectively 21.3% and 78.7%. The value of Li-Fe anti-site is 2.4%. Generally, Fe in Li vacancy sites significantly weakens the rate performance since the [010] direction is the exclusive pathway for lithium diffusion^19,34^. For R-LFP-Li_2_DHBN, the FePO_4_ phase was eliminated with a low Li-Fe anti-site value of 1.2% (Supplementary Table 7). This conclusion also explains the improved high-rate performance of the restored materials.

Although the above discussion reveals that the Li loss and the degraded FePO_4_ phase mainly exist at the surface of S-LFP particles, a deep understanding of failure mechanism in a single particle is still unclear. The electron energy loss spectroscopy (EELS) was applied to analyze the surface elemental valence state and concentration distribution of single particle (Fig. 4f, h). The Fe L_2,3_-edges are composed of two strong white lines due to the spin–orbit–split 2*p* core into unoccupied 3*d* state^42,43^. The energy loss shifts of Fe-L_2,3_ edges are a good indicator of changing Fe valence state. The O K-edge is originated from an excitation of the 1*s* orbital to unoccupied O 2*p* orbitals^44^. The Fe(III) phosphate has a clear pre-edge peak, whereas those with Fe(II) lack this feature, providing a characteristic of Fe valence state in LFP/FP. In the selected S-LFP particle-1 (Supplementary Fig. 22), Fe L_2,3_-edges gradually shift to a higher energy loss from point-1 to point-10, corresponding to the valence state change from Fe(II) to Fe(III) (Fig. 4g). The middle area is composed of two-phase coexistence (LFP + FP). The pre-edge peaks in O K-edge EELS also indicate the existence of Fe(III) near the center of the particle surface. Notably, Li K-edge peak exists at about 55 eV because a slight amount of Li^+^ still remains in the lithium-poor phase^45^. In the opposite scanning region of particle-1 (Supplementary Fig. 23), the Fe L-edge and O K-edge show a similar tendency, further proving that the selected particle surface is composed of FP near the center and LFP in the edge. The EELS results from another S-LFP particle-2 also show the same phase change (Supplementary Fig. 24), which is consistent with the previous reports of LFP cathode by refs. ^19,42^. However, not all particles in S-LFP electrode show the same characteristics. In another S-LFP particle-3, the Fe L_2,3_-edges locate at a higher energy loss, and the pre-edge peaks of O K-edge EELS are obvious from point-1 to point-10 (Supplementary Fig. 25). This indicates the only existence of FP phase throughout the surface of this particle. Based on the above discussion, it is concluded that the failure regions mainly occur at the surface in S-LFP cathode, which is also supported by the in-depth XPS results (Fig. 2o). Furthermore, the LFP/FP phase distribution is inhomogeneous at different places of each particle according to the EELS results. This feature is consistent with the phase evolution during charge and discharge process of LFP cathode^34,43,44^. By contrast, Fe L_2,3_-edges keep unchanged from point-1 to point-10 in R-LFP particle, and no pre-edge peak was detected in O K-edge EELS (Fig. 4i and Supplementary Fig. 26), suggesting that lithium ions have compensated into the vacancies and the Fe(III) phase has been eliminated after direct regeneration.

In total, the restoration mechanism of LFP is concluded as three points: (1) Componential compensation. The Li_2_DHBN couples well with the surface of the damaged LFP particles by its functional groups at a low temperature. High-temperature treatment enables Li-ions to enter the vacancies. (2) Structural restoration. The oxidation state of Fe(III) was reduced to its initial valence state, and Li-Fe anti-site value was also decreased, ascribed to the function of the cyano groups. (3) Improved reaction kinetics. The recoating of conductive carbon on the LFP particles is ascribed to the pyrolysis of Li_2_DHBN, which improves the Li-ion and electron transfer kinetics between the electrode and electrolyte.

### Techno-economic analysis of different battery recycling technologies

**Fig. 5:**
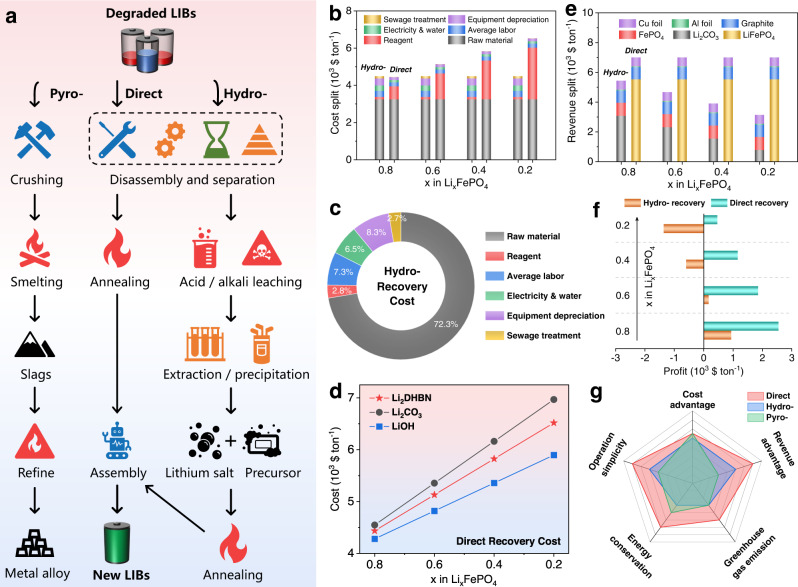
Techno-economic analysis of different battery recycling technologies. **a** Schematic of the pyro-, hydro- and direct routes. **b** Cost and **e** revenue splits based on the hydro- and direct recovery routes for different degraded LFP batteries. The residual lithium content indicates the degree of failure of the LFP cathode since this factor has a significant effect on the economic analysis. **c** Pie chart showing the cost proportions based on the hydro-recovery method. **d** Cost comparison on different lithium salts. **f** Profit calculations for the hydro- and direct recovery. **g** Comprehensive comparison of different battery recycling technologies.

Figure 5a summarizes the current end-of-life battery recycling technologies, including pyro-, hydro-, and direct routes. In this section, we shall mainly analyze the economic value and environmental benefit of the latter two technologies, assuming that 1 ton of degraded LFP batteries needs to be handled and 250 kg of degraded LFP powder is collected, according to the proportion of each component^46^. We assume that the residual lithium content is a critical factor in the degraded LFP batteries, which determines the cost of the different routes and the revenue they produce. For the hydro-route, a typical sulfur acid leaching technology was chosen as ref. ^47^. Figure 5b shows the different costs involved based on the real market price in China. The raw material (degraded LFP battery) cost is the main factor, accounting for about 72% of the total (Fig. 5c). In contrast, the other costs, including average labor, electricity and water, and equipment, are much higher for hydro- route due to the complex operational processes and the use of a strong acid. However, the residual lithium content is irrelevant in determining the total cost in hydro- route. For the direct route, the reagent cost (lithium salt) is a significant contributor to the overall cost (Supplementary Fig. 27), and increases with decreasing residual lithium content. The cost of using the three different lithium salts is also analyzed (Fig. 5d). The total cost of using the organic lithium salt is acceptable compared with traditional lithium salts.

As shown in Fig. 5e, the collected lithium carbonate and iron phosphate account for the main revenue in the hydro-route. The overall revenue is proportional to the residual lithium content, since the smaller content of residual lithium in the degraded LFP batteries, the lower production of the collected lithium salt in final products. For the direct route, the restored LFP material is the major component in the revenue, with a percentage of about 79% (Supplementary Fig. 28). The overall revenue is almost unchanged for different amounts of residual lithium. Hence, the profits produced by the hydro- and direct routes are respectively 945 and 2556 $ ton^−1^ when x = 0.8 in Li_x_FePO_4_ (Fig. 5f). A low amount of residual lithium results in a revenue loss for the hydro-route but a positive profit for the direct route, meaning that the battery recycling route reported here is advantageous in practice.

Energy consumption and greenhouse gas (GHG) emissions are also important considerations in developing a sustainable recycling system. The total energy consumption of pyro- and hydro-processes are respectively 12.14 and 19.57 MJ kg^−1^ cell (Supplementary Fig. 29a). The material and energy use is mainly attributed to the high-temperature smelting in pyro-process. In the hydro-process, 89.5% of energy consumption comes from strong acid leaching and metal extraction/precipitation steps. As comparison, only 3.16 MJ kg^−1^ cell is required for direct process. The total GHG emissions are respectively 1.96, 1.49, and 0.59 kg kg^−1^ cell for pyro-, hydro-, and direct recovery (Supplementary Fig. 29b). The above results show the advantages in environmental protection and energy conservation of direct recovery process due to the reduced material consumption and simplified operation procedures. Three different battery recycling technologies have been compared and the conclusions are summarized in Fig. 5g. The direct route has a significant advantage due to the high value of the final products, especially the restored LFP cathode material. The direct route is also superior in operation simplicity, avoiding the complex disposal procedures^48–50^. The direct route is therefore feasible for the future recycling industry.

However, some challenges still need to be overcome before this method finds large-scale use. First, it may be still a challenge to restore highly degraded cathodes by solid direct regeneration. Second, although our economic analysis provides a feasible reference, additional factors need to be considered in the real world, including the collection and classification of spent batteries, and the impact of transportation^13,51,52^. Third, a general recovery technology is urgently needed for different cathode materials with different degrees of degradation to satisfy the practical criteria on a large scale. Although there are challenges involved in using the technique on a large scale, we believe with an improvement in the recycling system of spent LIBs, advanced direct recycling technologies will have a great benefit.

## Discussion

In summary, a multifunctional organic lithium salt was shown to directly restore degraded LFP cathode. Both in-depth XPS and EELS results reveal that the degradation regions mainly exist at the surface of S-LFP particles, and the distribution of LFP/FP phase is inhomogeneous at different places of each particle. The degraded LFP particles are well coupled with the functional groups of the Li_2_DHBN, so that lithium fills vacancies and cyano groups create a reductive atmosphere to eliminate Fe(III) formation. At the same time, pyrolysis of the salt produces an amorphous conductive carbon layer that coats the LFP particles, enabling the restored LFP material to have good cycle stability and low-temperature performance. Additionally, the organic lithium salt also enables the restoration of the spent transition metal oxide-based cathodes. Techno-economic analysis indicates that this direct regeneration route is more competitive than other battery recycling technologies, with a potentially higher financial benefit. This organic lithium salt provides us with a unique idea to explore more different lithium sources for direct regeneration of degraded LIBs cathodes.

## Methods

### Chemicals and spent LIBs disassembly

The chemical reagents were 3,4-dihydroxybenzonitrile (AR, 99.0%), anhydrous tetrahydrofuran (THF anhydrous, AR, 99.0%), lithium hydride (LiH, AR, 99.0%), sodium chloride (NaCl, AR, 99.0%), lithium carbonate (Li_2_CO_3_, AR, 99.0%), lithium hydroxide (LiOH, AR, 99.0%), polyvinylidene fluoride (PVDF, AR, 99%), and N-methyl-pyrrolidone (NMP, AR, 99.0%). The type of spent LFP batteries used in the recovery process is cyclinder cell (18650, manufacturer: LG). The detailed parameters are listed in Supplementary Table 1. These batteries were first discharged by soaking them in a NaCl solution to ensure safety during disassembly. After drying, they were manually disassembled and separated into cathodes, anodes, separators, and shells. The cathode powder (S-LFP) was obtained after separation from the Al foils.

### Synthesis of Li_2_DHBN

The organic lithium salt (3,4-dihydroxybenzonitrile dilithium salt, Li_2_DHBN) was synthesized based on a previous study^53^. 1 g of 3,4-dihydroxybenzonitrile was added into 30 mL tetrahydrofuran at room temperature and stirred for 10 min to form a transparent solution. Then 0.118 g lithium hydride was introduced into above solution inside a glove box; after which the mixture was stirred for 15 h and a yellow solution was obtained. After filtration, a precipitate of Li_2_DHBN was dried at 150 °C under vacuum for 6 h. The real pictures during Li_2_DHBN synthesis are shown in Supplementary Fig. 5. It is worth noting that the whole synthesis process was carried out at room temperature, and no extra heating process was required and no polluting gases were produced.

### Regeneration process of LiFePO_4_ cathodes

For the regeneration of LiFePO_4_ (R-LFP), spent cathode powder was mixed with Li_2_DHBN and ground for 15 min in an agate mortar. The mixture was then heated at 5 °C min^−1^ to 800 °C where it was sintered for 6 h under an Ar/H_2_ atmosphere. Single-factor experiments were also conducted to determine the best temperature and amount of Li_2_DHBN used. The corresponding results are listed in Supplemental Information. For comparison, inorganic salts (Li_2_CO_3_ and LiOH) were used as lithium sources under the same experimental conditions. For the regeneration of LiCoO_2_ (R-LCO) or LiNi_0.5_Co_0.2_Mn_0.3_O_2_ (R-NCM), the spent powder and Li_2_DHBN were heated in a muffle furnace at 5°/min to 500 °C and sintered for 5 h, and heated and then at the same rate to 800 °C and sintered for 10 h. The other experimental conditions were the same as for R-LFP.

### Materials characterizations

The chemical composition of the spent and regenerated powders was determined by ICP-OES (Agilent ICPOES730, USA). The valence state of the surface elements was determined by X-ray photoelectron spectroscopy (XPS; Thermo Scientific K-Alpha, USA). The phase composition was determined by X-ray diffraction (XRD; Bruker D8 Advance, Germany) with Cu Kα radiation in the 2*θ* range 10–80°. The morphology and microstructure were observed by field-emission scanning electron microscopy (FESEM; Zeiss, S-3500N, Japan) and HRTEM (FEI Talos F200x, USA). A Thermogravimetric analyzer (TG; Mettler-Toledo TG2, Switzerland) coupled with a Fourier Transform Infrared spectrometer (Thermo Scientific IS50, USA) was used to analyze the decomposition of Li_2_DHBN. Temperature-dependent XRD was performed from 30 °C to 800 °C at a heating rate of 5 °C min^−1^ and then kept at 800 °C for 1 h. Electron energy loss spectroscopy (EELS; ThermoFischer Spectra 300, USA) was used to analyze the element valence states.

### Electrochemical performance tests

The working electrodes were fabricated with active materials, acetylene black, and PVDF binder in weight ratios of 8:1:1. A homogeneous slurry was formed in a NMP solution and then coated on aluminum foil. After drying at 60 °C for 12 h, the electrodes were cut into small plates with a mass loading of 2–3 mg cm^−2^. The assembly of CR2032 coin cells was conducted in a glove box and lithium metal, Celgard2325, and 1.0 M LiPF_6_ in ethylene carbonate/dimethyl carbonate/diethyl carbonate (EC:DMC:DEC = 1:1:1 in volume) was used as the counter electrode, separator, and electrolyte, respectively. Galvanostatic measurements were performed on a NEWARE battery testing system with a voltage range of 2.5–4.3 V vs. Li^+^/Li. The CV and electrochemical impedance spectroscopy (EIS) tests were made using an electrochemical workstation (BioLogic SP-150e).

### Economic analysis of different battery recycling technologies

We mainly focus on the economic analysis of S-LFP battery recycling (1 ton in total) by comparing the hydro- route with the direct route. The cost splits were divided into raw material, reagent, average labor, electricity and water, equipment depreciation, and sewage treatment. To estimate the possible benefits, we assume that any recycled component is valuable and compensates for the recycling cost. Since the value of the separator, electrolyte, and shell is hard to assess in a real process, these components are not included in our revenue analysis. The residual lithium content determines the consumption of lithium salt in the direct route and the value of the final products in the hydro- route. We regard the residual lithium content as a critical factor in the economic analysis. The detailed process-based cost and revenue models are listed in original data of techno-economic analysis (Supplementary Tables 12–19).

We have done a comprehensive life-cycle analysis and techno-economic analysis of pyro-, hydro-, and direct recycling processes based on the EverBatt 2020 model, which is developed by Argonne National Laboratory. The materials requirements for the pyro- and hydro-recycling processes are obtained from the EverBatt model, and that for direct recycling is calculated based on our lab (Supplementary Table 8). The life-cycle total energy use and total emission of three recycling processes are composed of material, energy, and process emission, as summarized in Supplementary Table 9. Cost and revenue analysis of different recycling processes are also based on the EverBatt model (Supplementary Tables 10, 11 and Supplementary Fig. 30).

## Supplementary information

Supplementary Information

## Data Availability

The datasets generated during and/or analyzed during the current study are available from the corresponding author upon reasonable request.

## References

[1] Melin HE (2021). Global implications of the EU battery regulation. Science.

[2] Liu J (2019). Pathways for practical high-energy long-cycling lithium metal batteries. Nat. Energy.

[3] Nitta N, Wu FX, Lee JT, Yushin G (2015). Li-ion battery materials: present and future. Mater. Today.

[4] Chen B (2021). Efficient reversible conversion between MoS_2_ and Mo/Na_2_S enabled by graphene-supported single-atom catalysts. Adv. Mater..

[5] Ma J (2022). Adaptable eutectic salt for the direct recycling of highly degraded layer cathodes. J. Am. Chem. Soc..

[6] Yang XG, Liu T, Wang CY (2021). Thermally modulated lithium iron phosphate batteries for mass-market electric vehicles. Nat. Energy.

[7] Takahashi M, Ohtsuka H, Akuto K, Sakurai Y (2005). Confirmation of long-term cyclability and high thermal stability of LiFePO_4_ in prismatic lithium-ion cells. J. Electrochem. Soc..

[8] Lepage D, Michot C, Liang G, Gauthier M, Schougaard SB (2011). A soft chemistry approach to coating of LiFePO_4_ with a conducting polymer. Angew. Chem. Int. Ed..

[9] Xiang JW (2019). Alkali-metal anodes: from lab to market. Joule.

[10] Fan E (2020). Sustainable recycling technology for li-ion batteries and beyond: challenges and future prospects. Chem. Rev..

[11] Harper G (2019). Recycling lithium-ion batteries from electric vehicles. Nature.

[12] Wang JX (2022). Efficient extraction of lithium from anode for direct regeneration of cathode materials of spent Li-ion batteries. ACS Energy Lett..

[13] Ciez RE, Whitacre JF (2019). Examining different recycling processes for lithium-ion batteries. Nat. Sustain..

[14] Qian GN (2022). Value-creating upcycling of retired electric vehicle battery cathodes. Cell Rep. Phys. Sci..

[15] Jiao ML (2022). Recycling spent LiNi_1-x-y_Mn_x_Co_y_O_2_ cathodes to bifunctional NiMnCo catalysts for zinc-air batteries. Proc. Natl Acad. Sci. USA.

[16] Fan M (2021). Increased residual lithium compounds guided design for green recycling of spent lithium-ion cathodes. Energy Environ. Sci..

[17] Wang JX (2022). Direct conversion of degraded LiCoO_2_ cathode materials into high-performance LiCoO_2_: a closed-loop green recycling strategy for spent lithium-ion batteries. Energy Storage Mater..

[18] Ma XT (2021). Recycled cathode materials enabled superior performance for lithium-ion batteries. Joule.

[19] Xu PP (2020). Efficient direct recycling of lithium-ion battery cathodes by targeted healing. Joule.

[20] Park KY (2014). Anti-site reordering in LiFePO_4_: Defect annihilation on charge carrier injection. Chem. Mater..

[21] Fan M (2022). In situ electrochemical regeneration of degraded LiFePO_4_ electrode with functionalized prelithiation separator. Adv. Energy Mater..

[22] Liu X (2022). Direct regeneration of spent lithium iron phosphate via a low-temperature molten salt process coupled with a reductive environment. Ind. Eng. Chem. Res..

[23] Jing QK (2020). Direct regeneration of spent LiFePO_4_ cathode material by a green and efficient one-step hydrothermal method. ACS Sustain. Chem. Eng..

[24] Song W (2019). Re-synthesis of nano-structured LiFePO_4_/graphene composite derived from spent lithium-ion battery for booming electric vehicle application. J. Power Sources.

[25] Ji GJ (2021). Efficient utilization of scrapped l LiFePO_4_ battery for novel synthesis of Fe_2_P_2_O_7_/C as candidate anode materials. Resour. Conserv. Recycl..

[26] Hu LH, Wu FY, Lin CT, Khlobystov AN, Li LJ (2013). Graphene-modified LiFePO_4_ cathode for lithium ion battery beyond theoretical capacity. Nat. Commun..

[27] Hu YS (2007). Improved electrode performance of porous LiFePO_4_ using RuO_2_ as an oxidic nanoscale interconnect. Adv. Mater..

[28] Li XL, Zhang J, Song DW, Song JS, Zhang LQ (2017). Direct regeneration of recycled cathode material mixture from scrapped LiFePO_4_ batteries. J. Power Sources.

[29] Sun QF (2020). Resynthesizing LiFePO_4_/C materials from the recycled cathode via a green full-solid route. J. Alloy. Compd..

[30] Song YF (2021). Regeneration of LiFePO_4_ from spent lithium-ion batteries via a facile process featuring acid leaching and hydrothermal synthesis. Green. Chem..

[31] Wu XL (2013). Carbon-nanotube-decorated nano-LiFePO_4_ @C cathode material with superior high-rate and low-temperature performances for lithium-ion batteries. Adv. Energy Mater..

[32] Huang GL (2013). Polyvinylpyrrolidone (PVP) assisted synthesized nano-LiFePO_4_/C composite with enhanced low-temperature performance. Electrochim. Acta.

[33] Jia, K. et al. Long-life regenerated LiFePO4 from spent cathode by elevating the *d*-band center of Fe. *Adv. Mater*. 2208034 (2022). 10.1002/adma.202208034 36300803

[34] Hong L (2017). Two-dimensional lithium diffusion behavior and probable hybrid phase transformation kinetics in olivine lithium iron phosphate. Nat. Commun..

[35] Wang R (2021). Twin boundary defect engineering improves lithium-ion diffusion for fast-charging spinel cathode materials. Nat. Commun..

[36] Zhang JF (2019). Enhancing high-voltage performance of Ni-rich cathode by surface modification of self-assembled nasicon fast ionic conductor LiZr_2_(PO_4_)_3_. ACS Appl. Mater. Interfaces.

[37] Jia K (2022). Suppressed lattice oxygen release via Ni/Mn doping from spent LiNi_0.5_Mn_0.3_Co_0.2_O_2_ toward high-energy layered-oxide cathodes. Nano Lett..

[38] Zhou L (2022). High areal capacity, long cycle life 4 V ceramic all-solid-state li-ion batteries enabled by chloride solid electrolytes. Nat. Energy.

[39] Zhang JF (2020). Construction of TiP_2_O_7_ nanosheets/rGO hierarchical flower-like heterostructures for superfast and ultralong lithiation/delithiation process. Appl. Surf. Sci..

[40] Oswald S, Pritzl D, Wetjen M, Gasteiger HA (2020). Novel method for monitoring the electrochemical capacitance by in situ impedance spectroscopy as indicator for particle cracking of nickel-rich NCMs: Part I. Theory and validation. J. Electrochem. Soc..

[41] Zhang YR (2020). Revealing electrolyte oxidation via carbonate dehydrogenation on Ni-based oxides in Li-ion batteries by in situ Fourier transform infrared spectroscopy. Energy Environ. Sci..

[42] Laffont L (2006). Study of the LiFePO_4_/FePO_4_ two-phase system by high-resolution electron energy loss spectroscopy. Chem. Mater..

[43] Li D, Zhou HS (2014). Two-phase transition of Li-intercalation compounds in Li-ion batteries. Mater. Today.

[44] Boesenberg U (2013). Mesoscale phase distribution in single particles of LiFePO_4_ following lithium deintercalation. Chem. Mater..

[45] Lachal M (2017). Remarkable impact of grains boundaries on the chemical delithiation kinetics of LiFePO_4_. Solid State Ion..

[46] Jung JCY, Sui PC, Zhang JJ (2021). A review of recycling spent lithium-ion battery cathode materials using hydrometallurgical treatments. J. Storage Mater..

[47] Li H (2017). Recovery of lithium, iron, and phosphorus from spent LiFePO_4_ batteries using stoichiometric sulfuric acid leaching system. ACS Sustain. Chem. Eng..

[48] Wang T (2020). Direct recycling of spent NCM cathodes through ionothermal lithiation. Adv. Energy Mater..

[49] Piątek J (2020). Sustainable Li‐ion batteries: chemistry and recycling. Adv. Energy Mater..

[50] Mrozik W, Rajaeifar MA, Heidrich O, Christensen P (2021). Environmental impacts, pollution sources and pathways of spent lithium-ion batteries. Energy Environ. Sci..

[51] Lander L (2021). Financial viability of electric vehicle lithium-ion battery recycling. iScience.

[52] Wang JX (2022). Direct and green repairing of degraded LiCoO_2_ for reuse in lithium-ion batteries. Natl Sci. Rev..

[53] Jezowski P (2018). Safe and recyclable lithium-ion capacitors using sacrificial organic lithium salt. Nat. Mater..

