# Supplementary material for: Direct regeneration of degraded lithium-ion battery cathodes with a multifunctional organic lithium salt
Source: Nat Commun. 2023 Feb 3;14:584. doi: 10.1038/s41467-023-36197-6 (PMC9898549; doi:10.1038/s41467-023-36197-6)
Supplement: Supplementary file 1 — Supplementary Information [file 41467_2023_36197_MOESM1_ESM.pdf]

Supplementary Information for

Direct regeneration of degraded lithium-ion battery cathodes with a multifunctional organic lithium salt

Guanjun Ji[1,2#], Junxiong Wang[1,2#], Zheng Liang[2*], Kai Jia[1,2], Jun Ma[1], Zhaofeng Zhuang[1], Guangmin Zhou[1*], Hui-Ming Cheng[3,4*]

[1] *Tsinghua-Berkeley Shenzhen Institute & Tsinghua Shenzhen International Graduate School, Tsinghua University, Shenzhen 518055, China*

[2] *Frontiers Science Center for Transformative Molecules, School of Chemistry and Chemical Engineering, Shanghai Jiao Tong University, Shanghai 200240, China*

[3] *Faculty of Materials Science and Engineering / Institute of Technology for Carbon Neutrality, Shenzhen Institute of Advanced Technology, Chinese Academy of Science, Shenzhen 518055, China*

[4] *Shenyang National Laboratory for Materials Science, Institute of Metal Research, Chinese Academy of Sciences, Shenyang 110016, China*

* Corresponding authors: Zheng Liang (Email: liangzheng06@sjtu.edu.cn); Guangmin Zhou (Email: guangminzhou@sz.tsinghua.edu.cn); Hui-Ming Cheng (Email: cheng@imr.ac.cn)

# These authors contributed equally to this work.

This file includes:

Supplementary Text

Supplementary Fig. 1 to Fig. 30

Supplementary Table 1 to Table 19

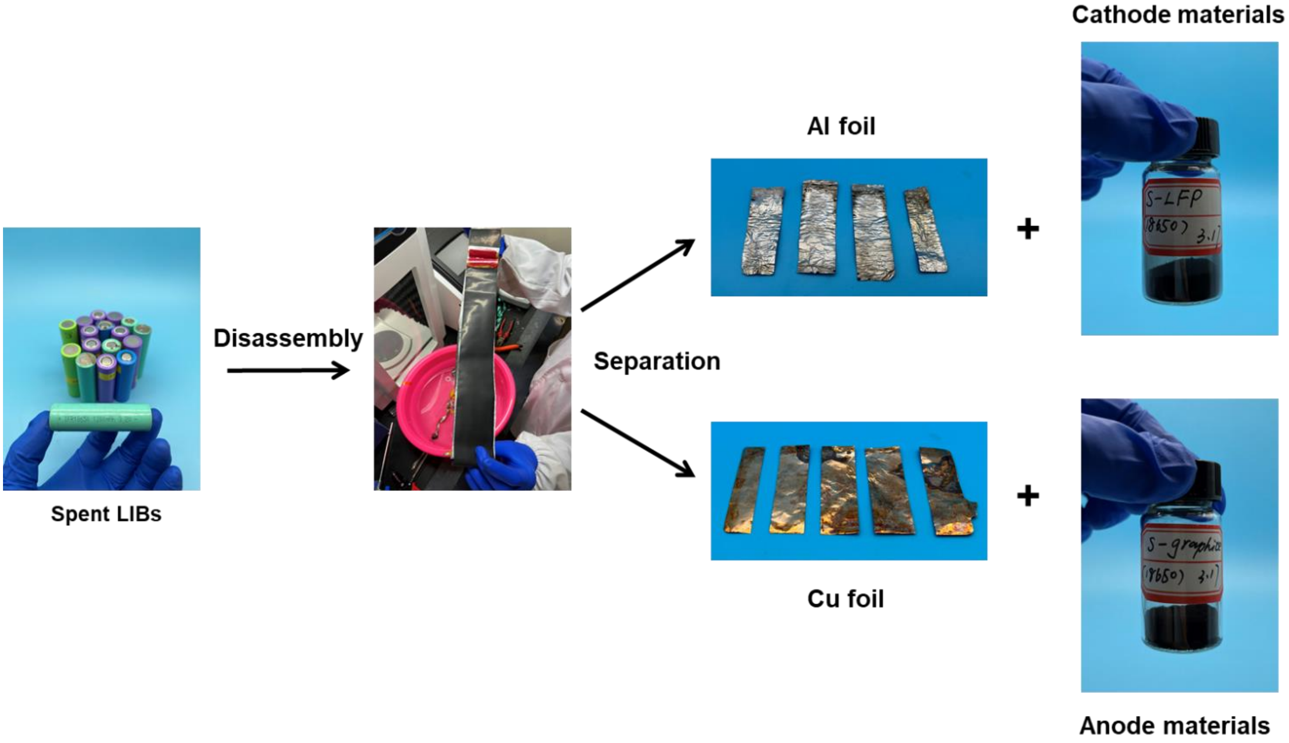

Supplementary Fig. 1 Recycling procedure of spent LIBs. 18650 degraded LIBs (1.2 Ah) were manually disassembled in a fume cupboard and the cathodes, anodes, and separators removed. The cathode sheets were immersed in water and the spent powder could then be easily separated from the Al foil due to its water-based binder. For the anode sheets, spent graphite powder was also collected during the same operation.

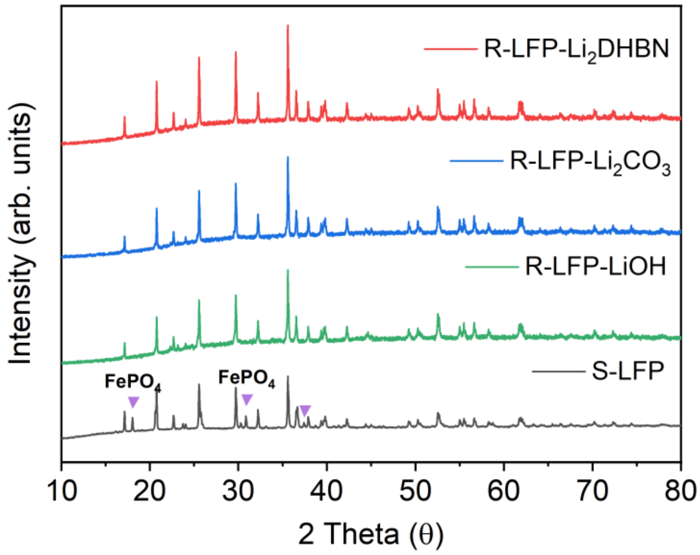

Supplementary Fig. 2 XRD spectra of S-LFP, R-LFP-LiOH, R-LFP- Li_2CO_3, and R-LFP-Li_2DHBN.

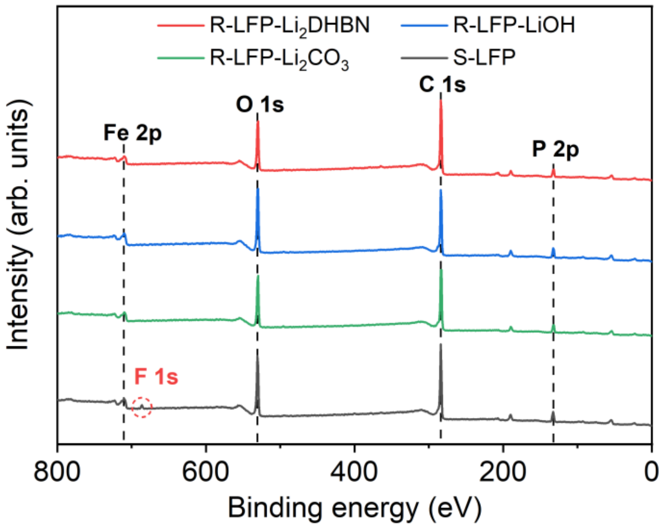

Supplementary Fig. 3 XPS survey of S-LFP, R-LFP-LiOH, R-LFP-Li_2CO_3, and R-LFP-Li_2DHBN.

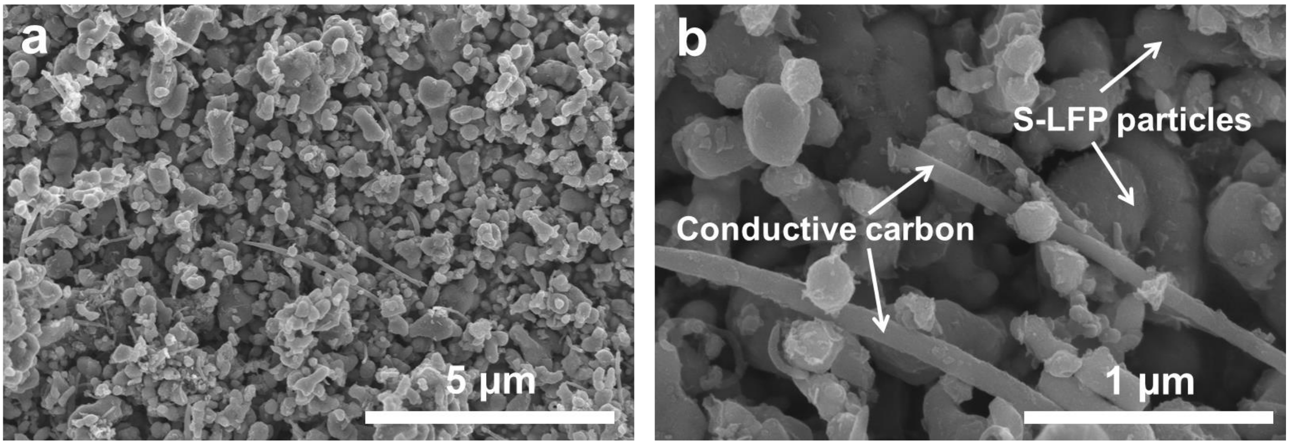

Supplementary Fig. 4 SEM images of degraded LFP cathode materials at **a** low and **b** high resolutions.

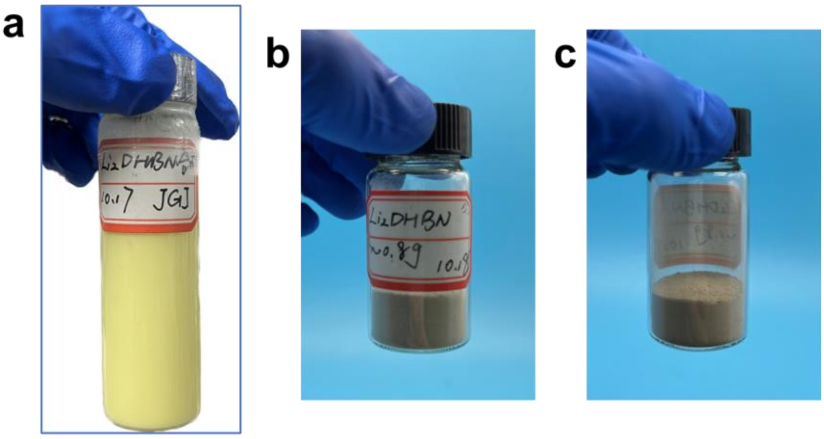

Supplementary Fig. 5 The pictures during Li_2DHBN synthesis process. **a** A yellow solution before filtration. **b, c** The final Li_2DHBN precipitate after filtration and dry.

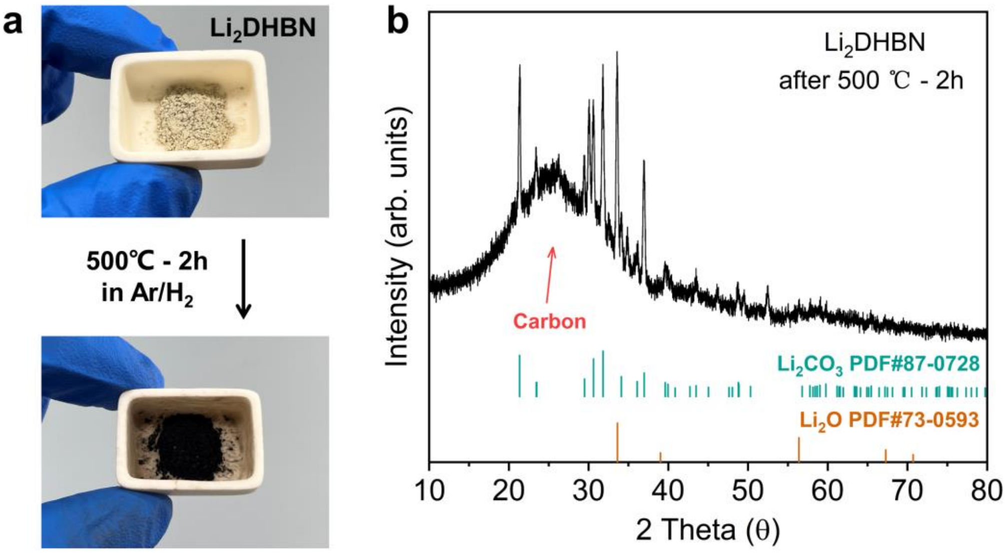

Supplementary Fig. 6 a The pictures of Li_2DHBN before and after heat treatment. **b** XRD spectra of Li_2DHBN after heat treatment.

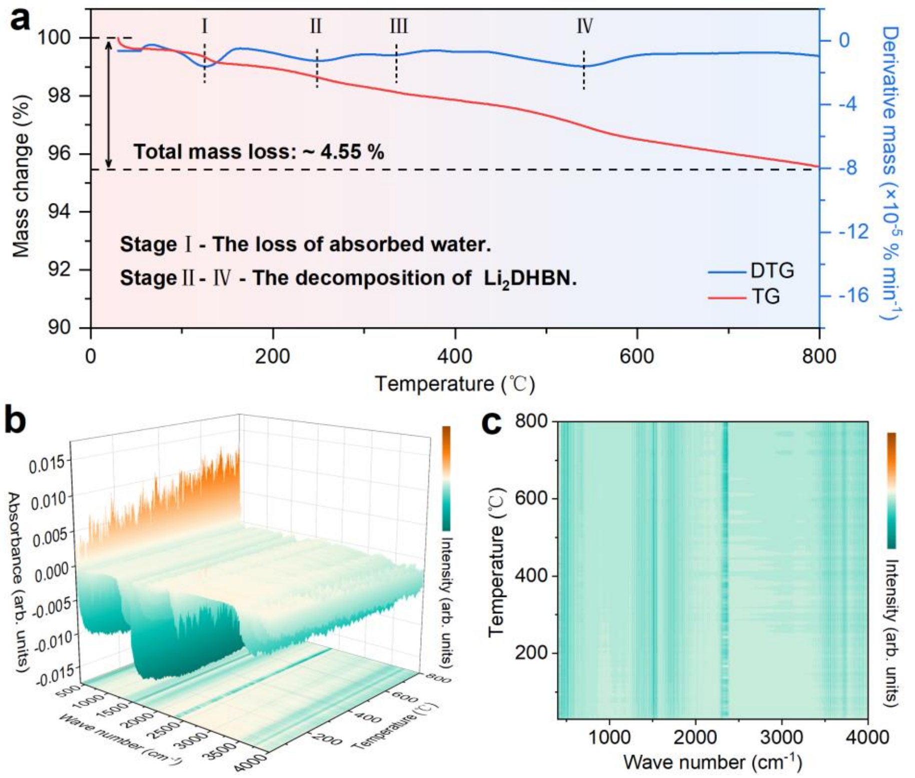

Supplementary Fig. 7 a TG-DTA, **b** TG-IR, and **c** IR contour map of the mixture of S-LFP powder and Li_2DHBN. To simulate our experimental process, the mixture of S-LFP powder and Li_2DHBN was also used for TR-FTIR measurements. The optimum weight of organic lithium salt is 5 wt%. The peaks are not obvious from the IR contour map due to the small quantity of Li_2DHBN. TG-DTA results show the same decomposition process as **Fig. 1e**.

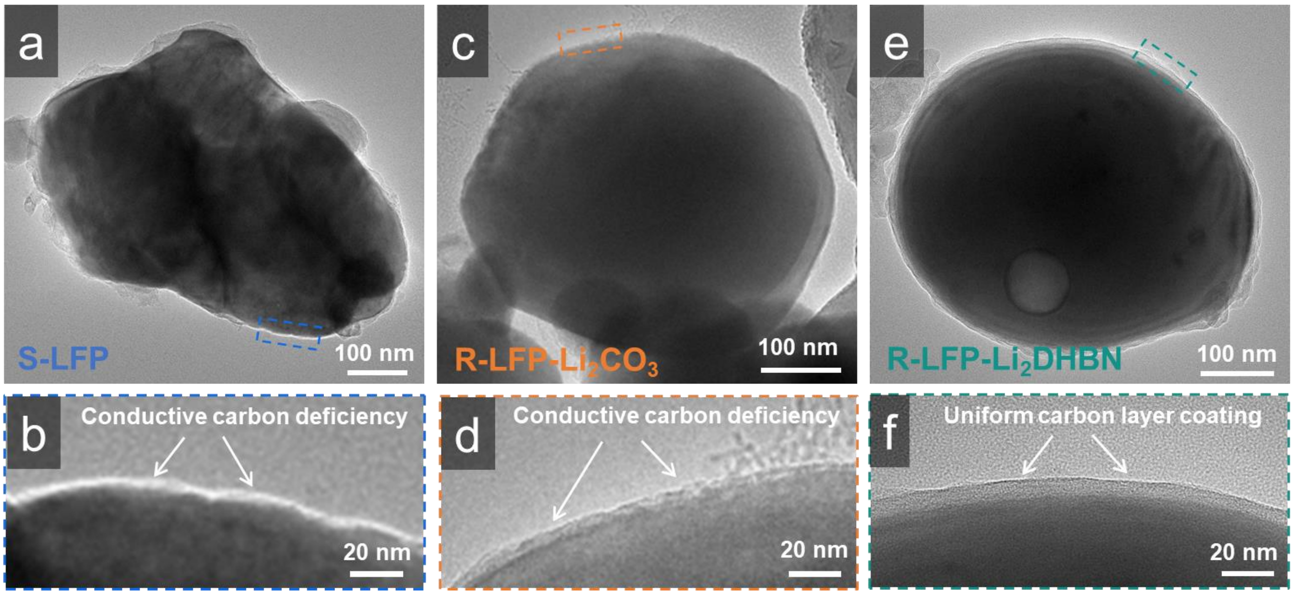

Supplementary Fig. 8 TEM images and corresponding enlarged figures of **a, b** S-LFP, **c, d** R-LFP-Li_2CO_3, and **e, f** R-LFP-Li_2DHBN.

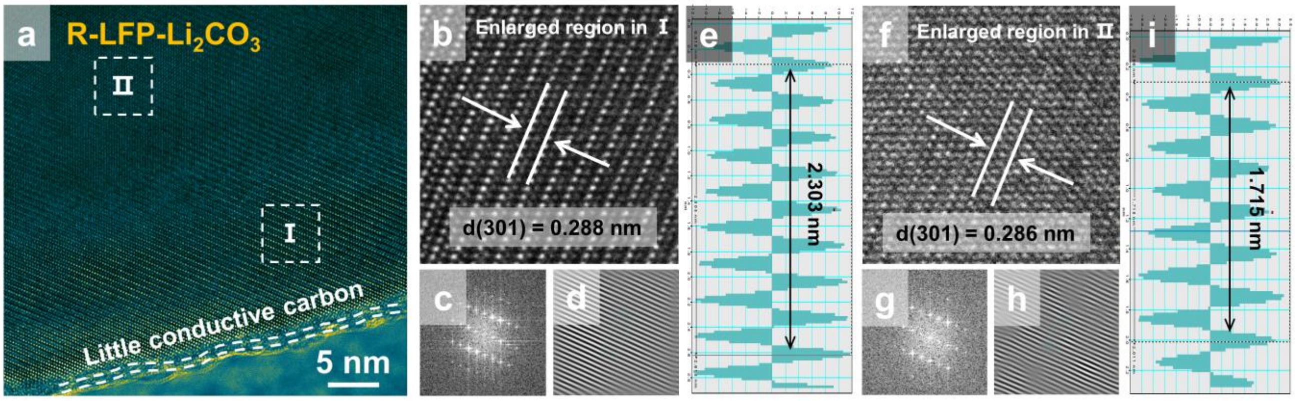

Supplementary Fig. 9 Microstructure characterization of R-LFP-Li_2CO_3 cathode. a TEM image, **b, f** enlarged figures, **c, g** SAED images from FFT results, **d, h** inverse FFT results, and **e, i** the corresponding line profiles in Supplementary Figs. 9d and 9h.

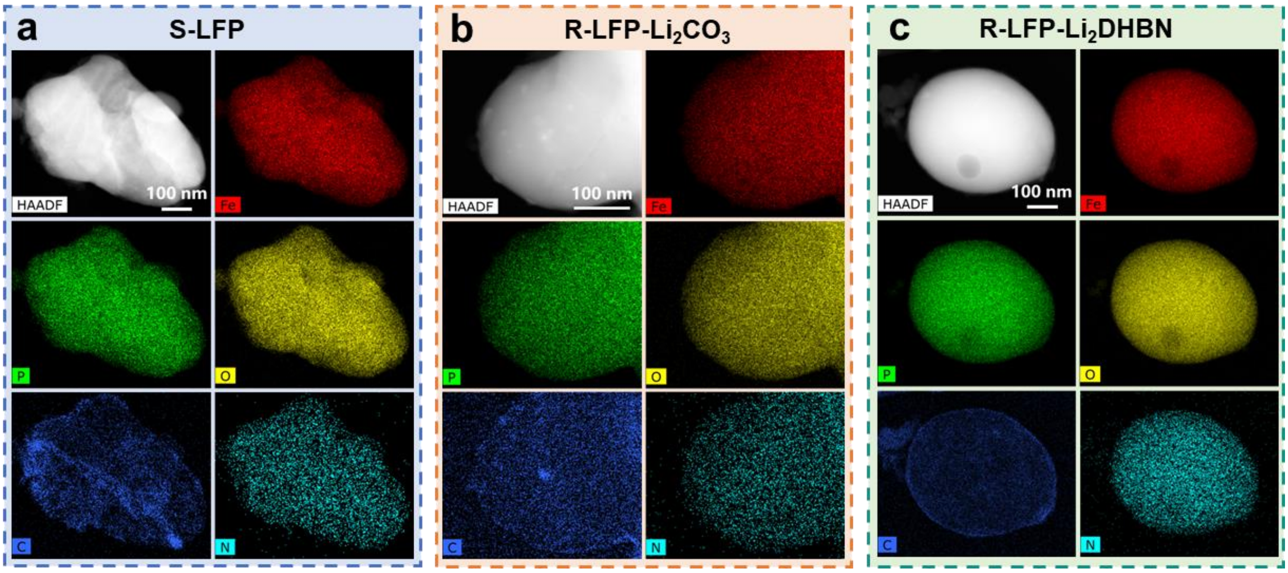

Supplementary Fig. 10 EDS elemental maps of **a** S-LFP, **b** R-LFP-Li_2CO_3 and **c** R-LFP-Li_2DHBN.

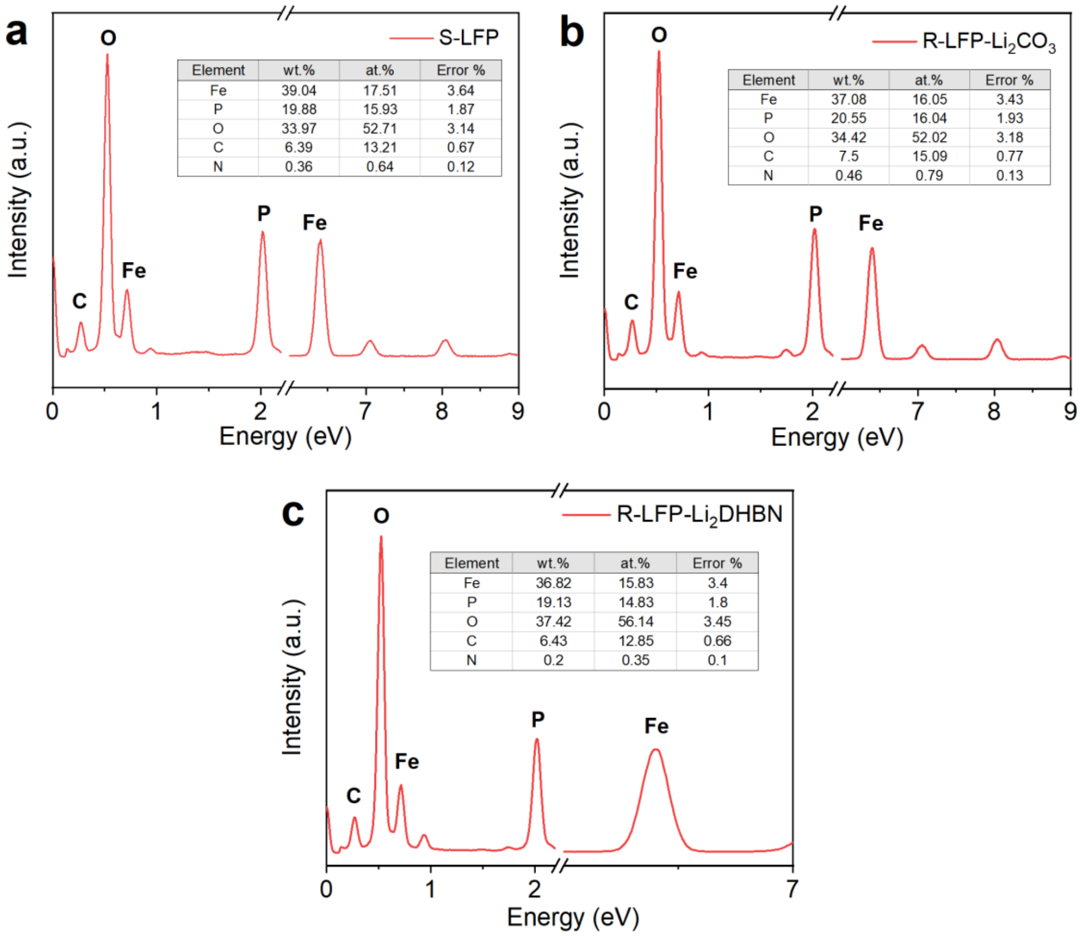

Supplementary Fig. 11 EDS energy spectra of **a** S-LFP, **b** R-LFP-Li_2CO_3 and **c** R-LFP-Li_2DHBN.

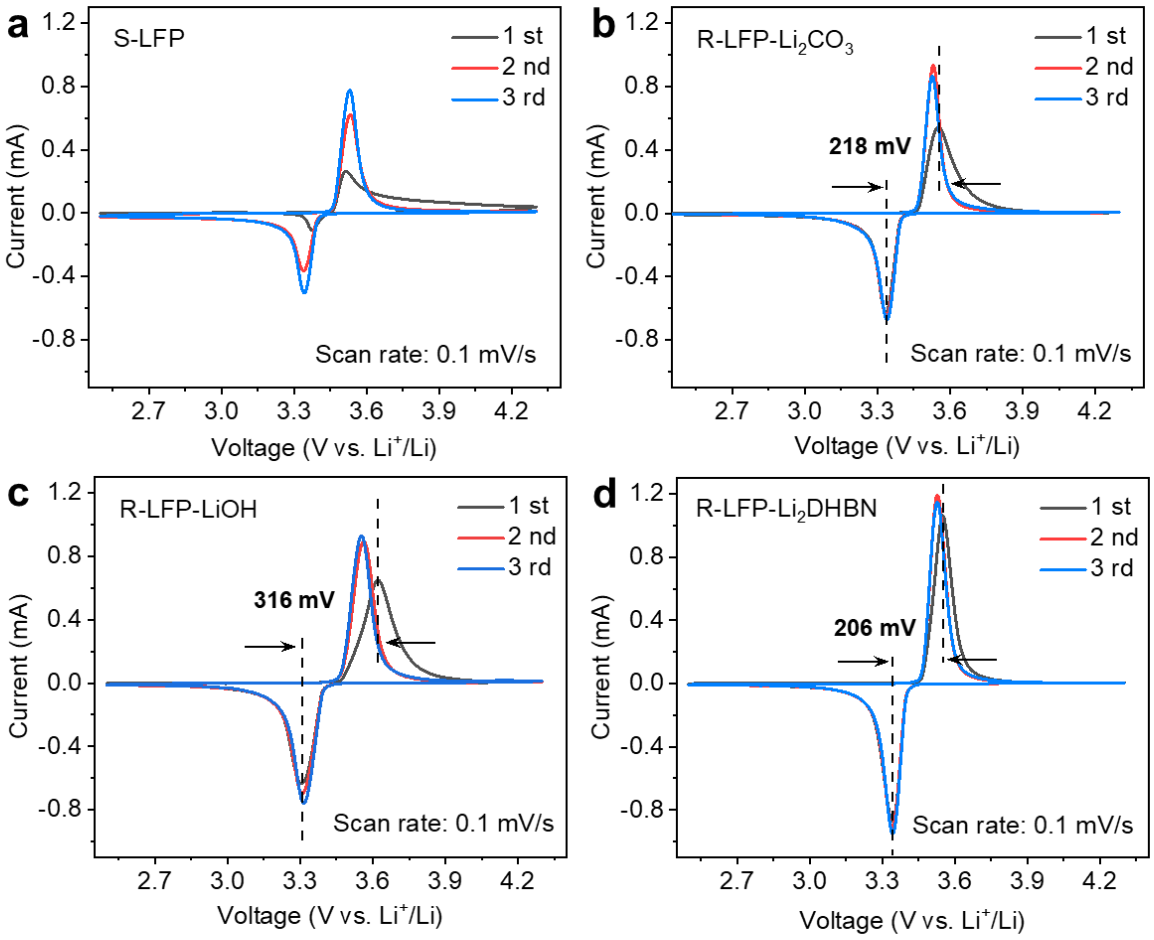

Supplementary Fig. 12 The initial three CV curves of **a** S-LFP, **b** R-LFP-LiOH, **c** R-LFP-Li_2CO_3, **d** R-LFP-Li_2DHBN.

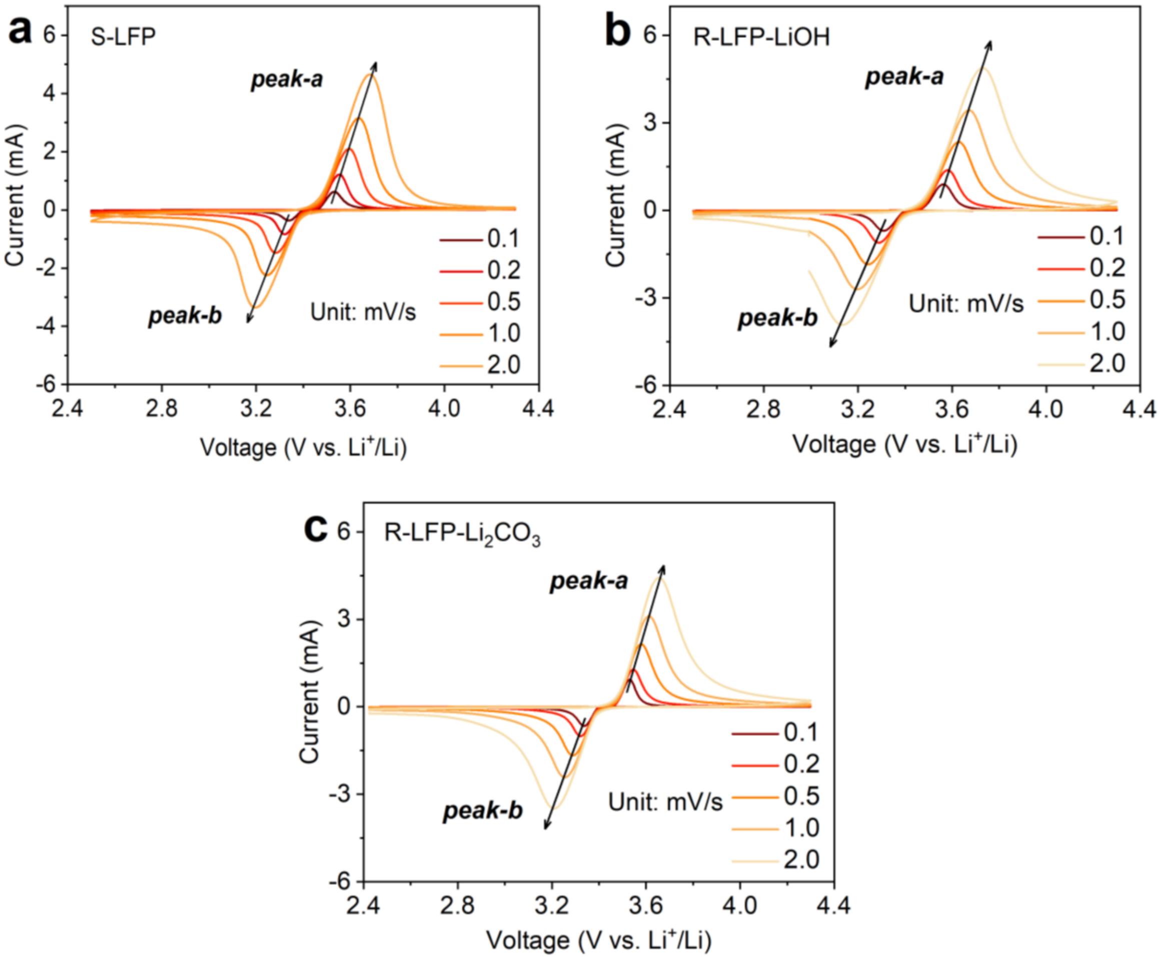

Supplementary Fig. 13 CV curves at different scan rates of **a** S-LFP, **b** R-LFP-LiOH, and **c** R-LFP-Li_2CO_3.

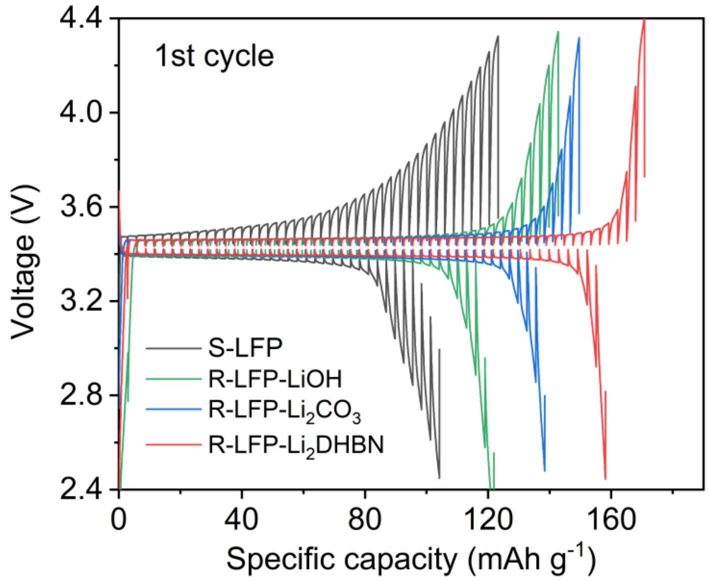

Supplementary Fig. 14 GITT curves during the first cycle of S-LFP, R-LFP-LiOH, R-LFP- Li_2CO_3, and R-LFP-Li_2DHBN.

The Li-ion diffusion rate calculated from the GITT curve is based on the following formula according to Fick's second law of diffusion[1, 2, 3]:

$D=4/\pi\tau\ (n_M V_M/S)^2(\Delta E_S/\Delta E_t)^2$

where τ is the duration of the current pulse, n_M is the number of moles, V_M is the molar volume of the electrode, S is the electrode-electrolyte contact area, and ΔE_S and ΔE_t are the changes in the steady state potential and the total change during the current flux by deducting the IR drop, respectively. In our experiments, τ = 1800 s, n_M = m/M_{LFP}, M_{LFP} = 157.76 g mol^{-1}, V_M = 20.5 cm^3 mol^{-1}, S = 1.13 cm^3.

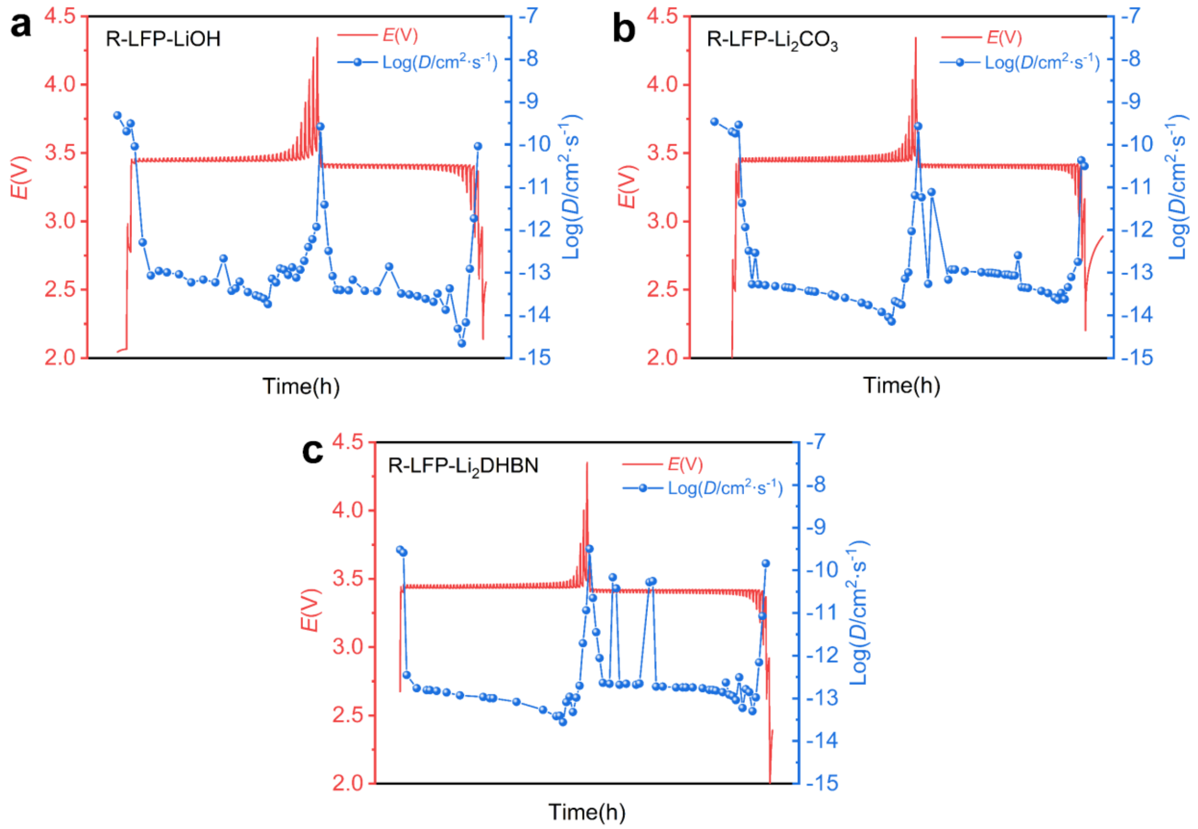

Supplementary Fig. 15 GITT profiles for the discharge/charge process (red curves) and diffusion coefficients (blue curves) of **a** R-LFP-LiOH, **b** R-LFP- Li_2CO_3, and **c** R-LFP-Li_2DHBN.

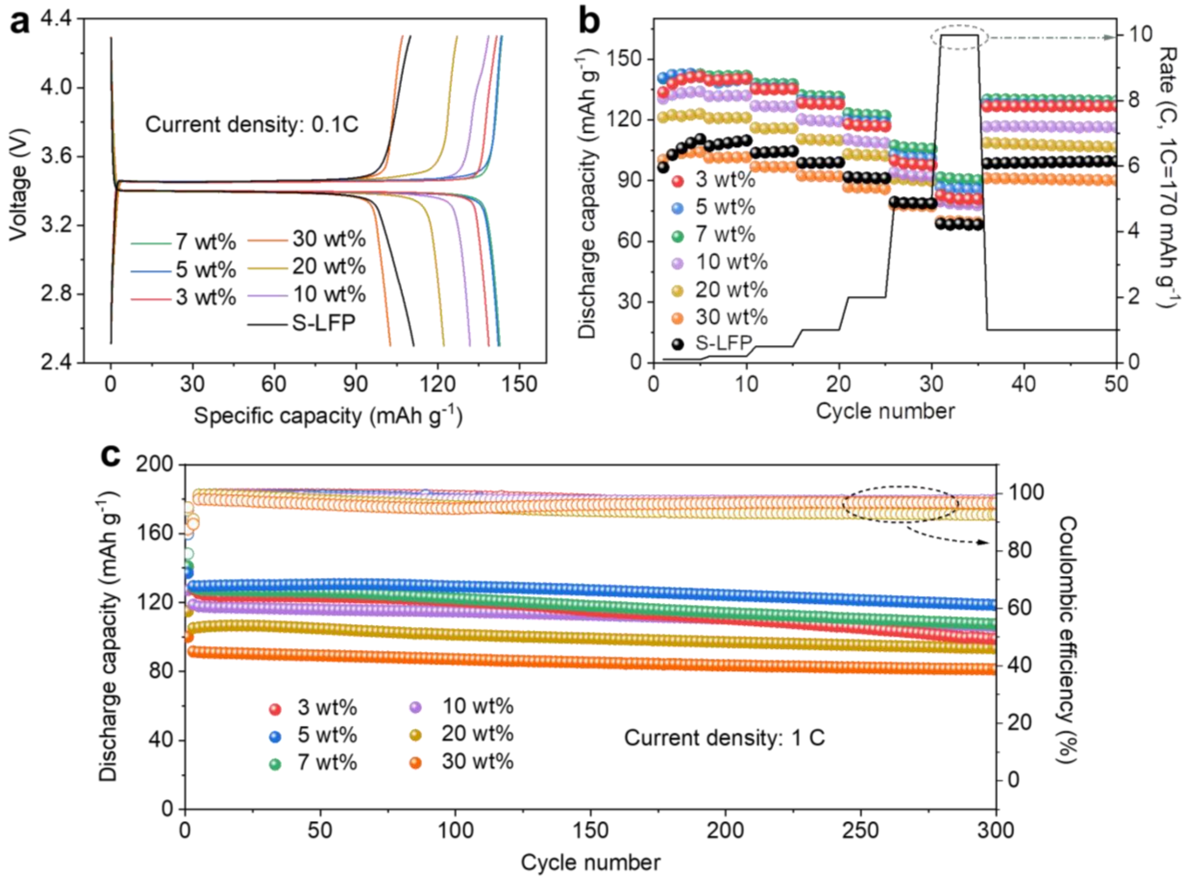

Supplementary Fig. 16 The electrochemical performance obtained from different usages of Li_2DHBN. **a** Charge-discharge curves, **b** rate capabilities, **c** cycling performance of R-LFP-Li_2DHBN.

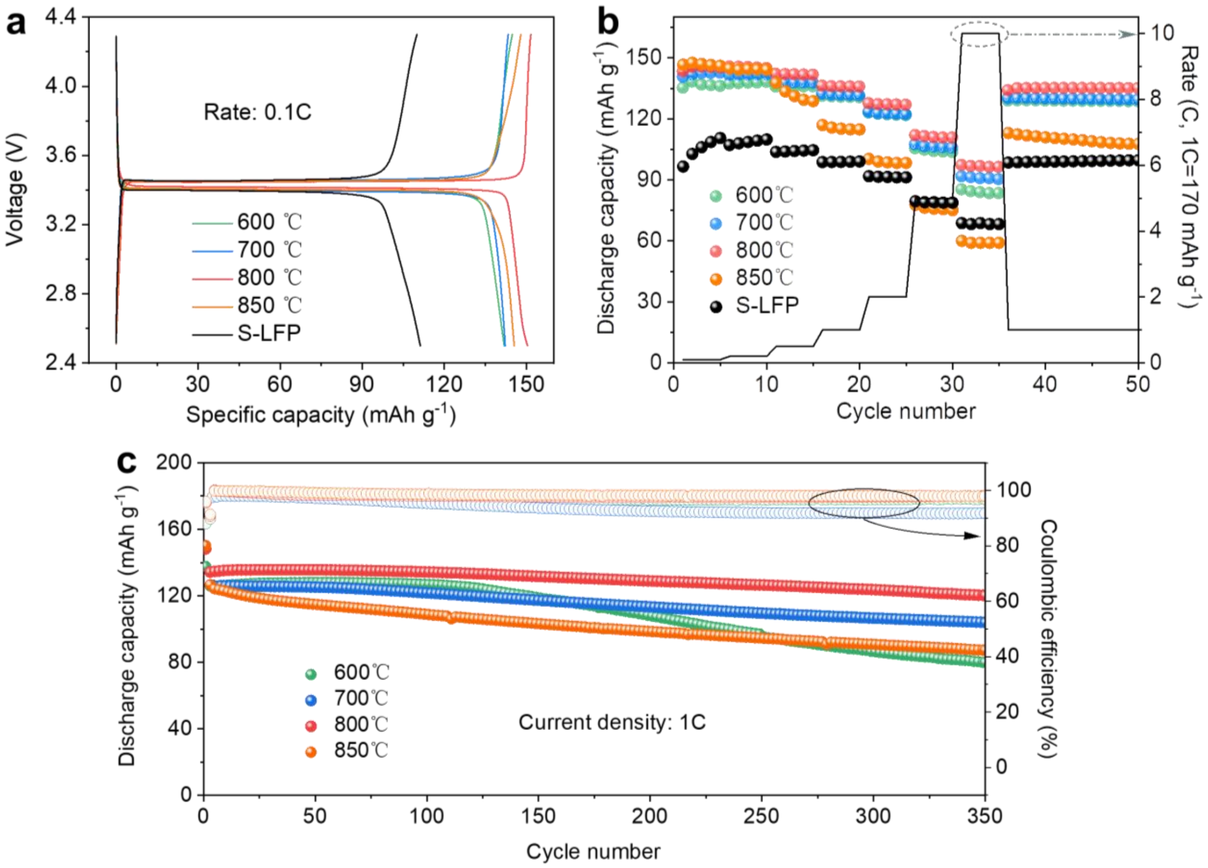

Supplementary Fig. 17 The electrochemical performance obtained at different temperatures. **a** Charge-discharge curves, **b** rate capabilities, **c** cycling performance of R-LFP-Li_2DHBN.

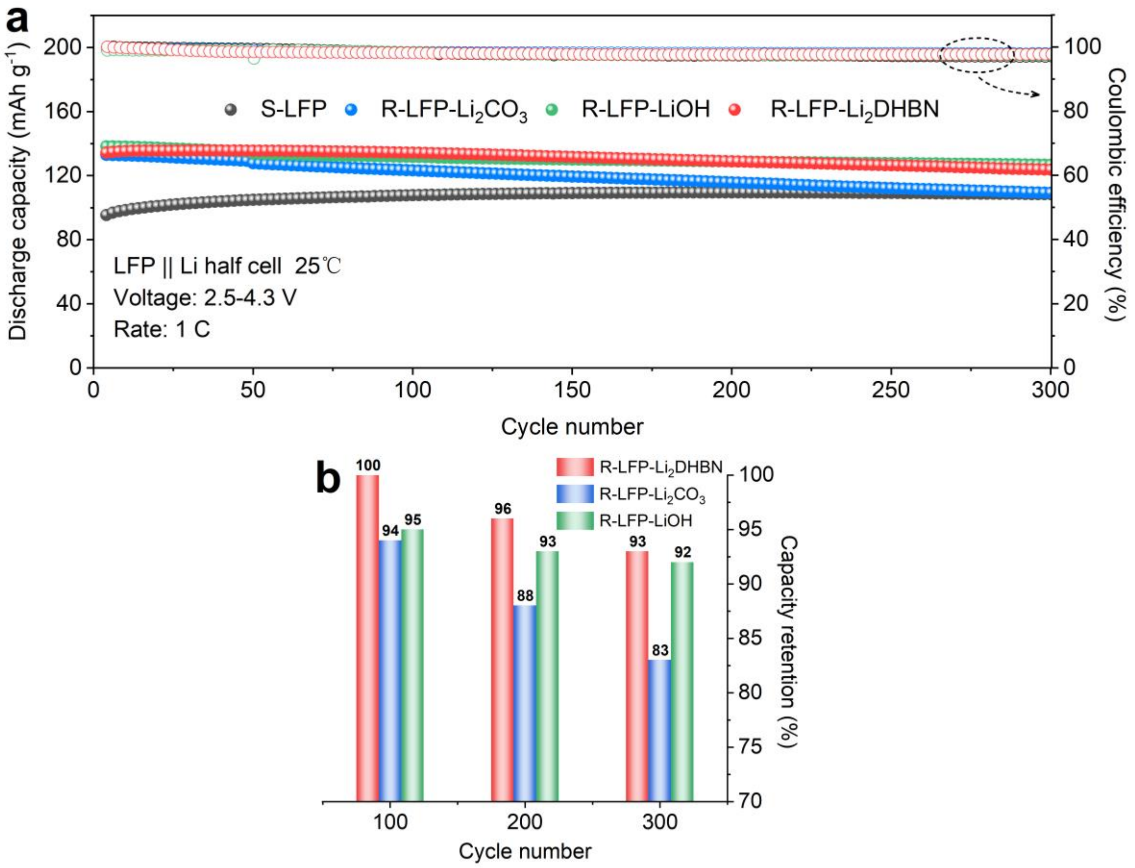

Supplementary Fig. 18 a The cycling performance at 1 C rate and **b** the corresponding capacity retentions of S-LFP, R-LFP-LiOH, R-LFP- Li_2CO_3, and R-LFP-Li_2DHBN.

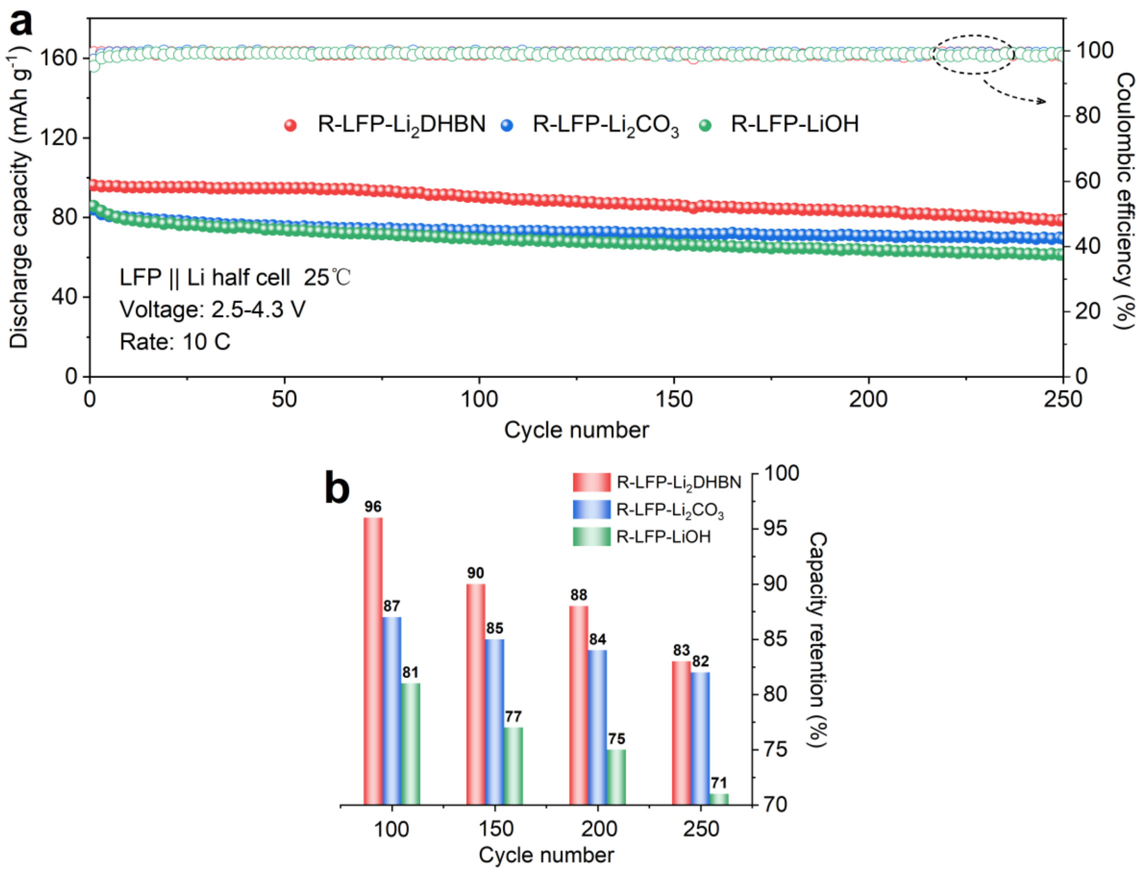

Supplementary Fig. 19 a The cycling performance at 10 C rate and **b** the corresponding capacity retentions of R-LFP-LiOH, R-LFP- Li_2CO_3, and R-LFP-Li_2DHBN.

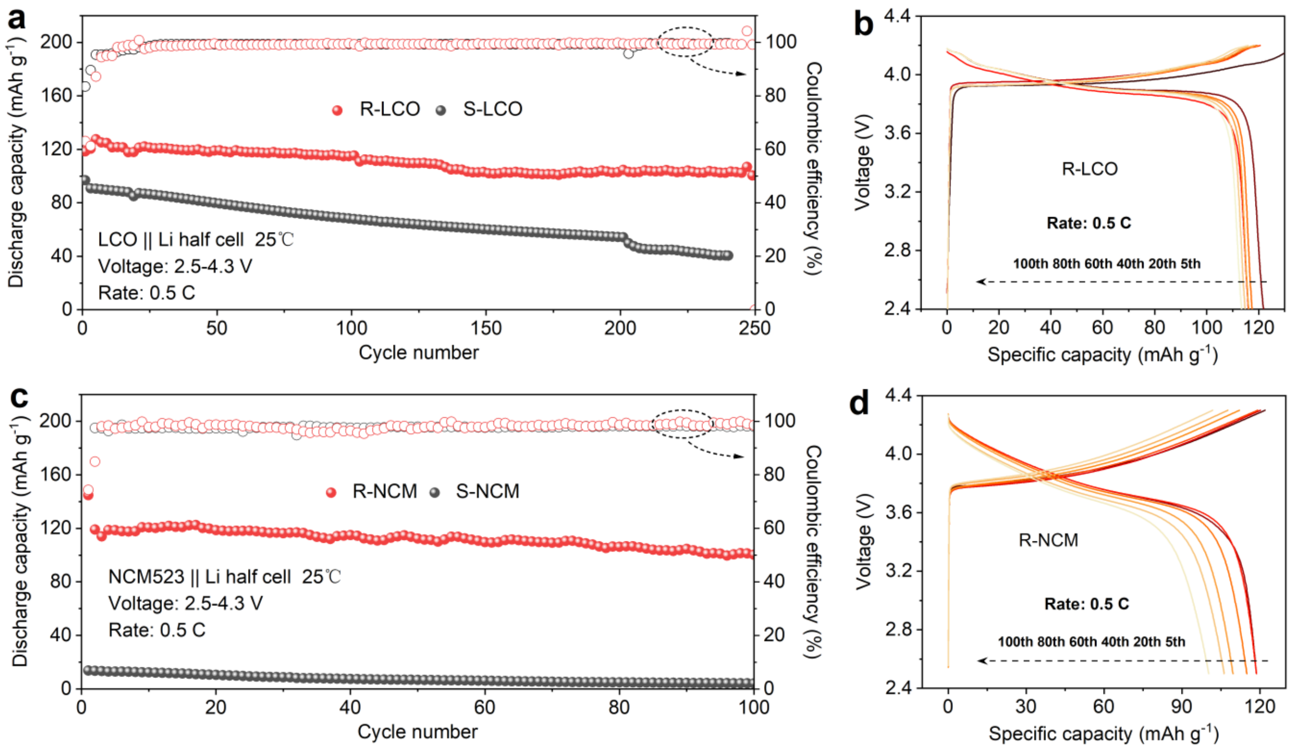

Supplementary Fig. 20 Demonstration of the versatility of Li_2DHBN. The cycling performance and charge-discharge profiles at a rate of 0.5 C of **a, b** R-NCM and **c, d** R-LCO.

To demonstrate the versatility of the organic lithium salt, S-LCO and S-NCM cathodes were also investigated under the optimal experimental conditions. As depicted in **Supplementary Fig. 20**, the R-LCO sample has a specific discharge capacity of 125 mAh g^{-1} after 250 cycles with a high retention of 83%. In comparison, the S-LCO could only retain a capacity of 90 mAh g^{-1} which decreased sharply after 200 cycles. For the R-NCM sample, the improved specific capacity reached about 120 mAh g^{-1} and retained 84% of the initial value. The original S-NCM sample was almost completely degraded after long cycling. It is concluded that the Li_2DHBN can be used to restore different types of spent LIB cathodes with different degrees of degradation, thus providing a competitive lithium supplement for future direct regeneration technology.

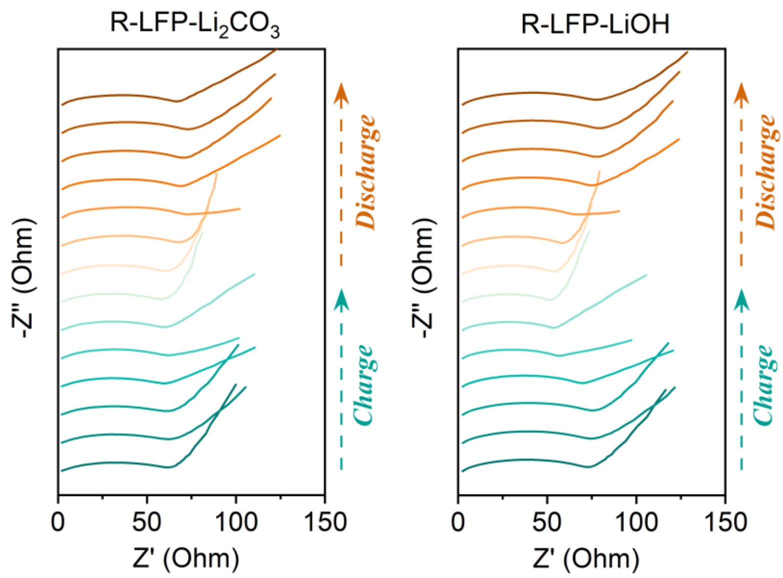

Supplementary Fig. 21 The impedance spectra of **a** R-LFP-Li_2CO_3 and **b** R-LFP-LiOH collected during the first cycle.

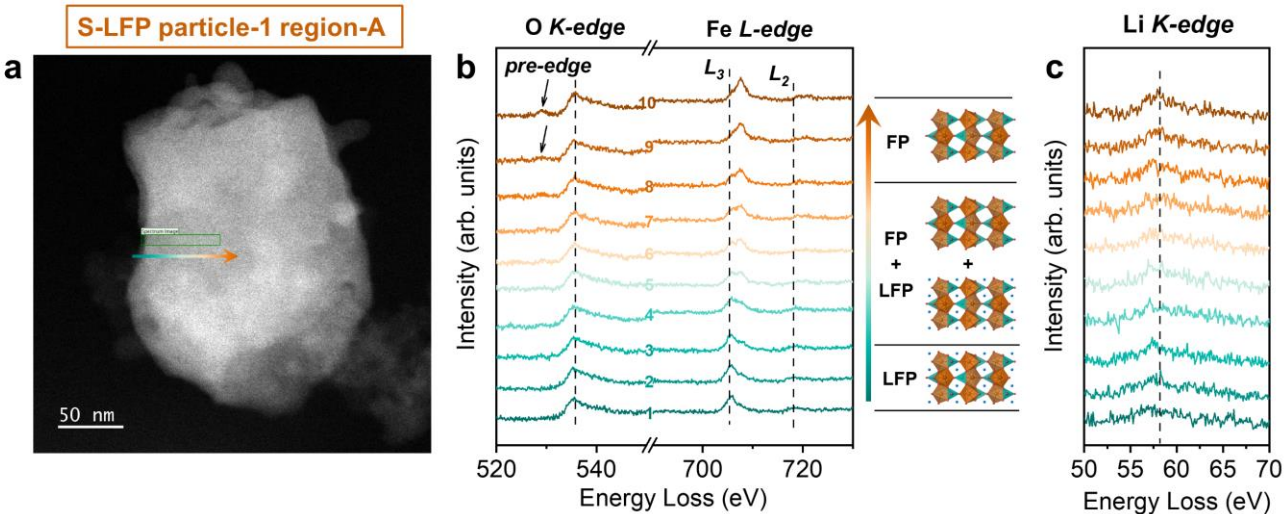

Supplementary Fig. 22 a STEM image, **b** O K-edge and Fe L-edge, and **c** Li K-edge EELS of S-LFP particle-1.

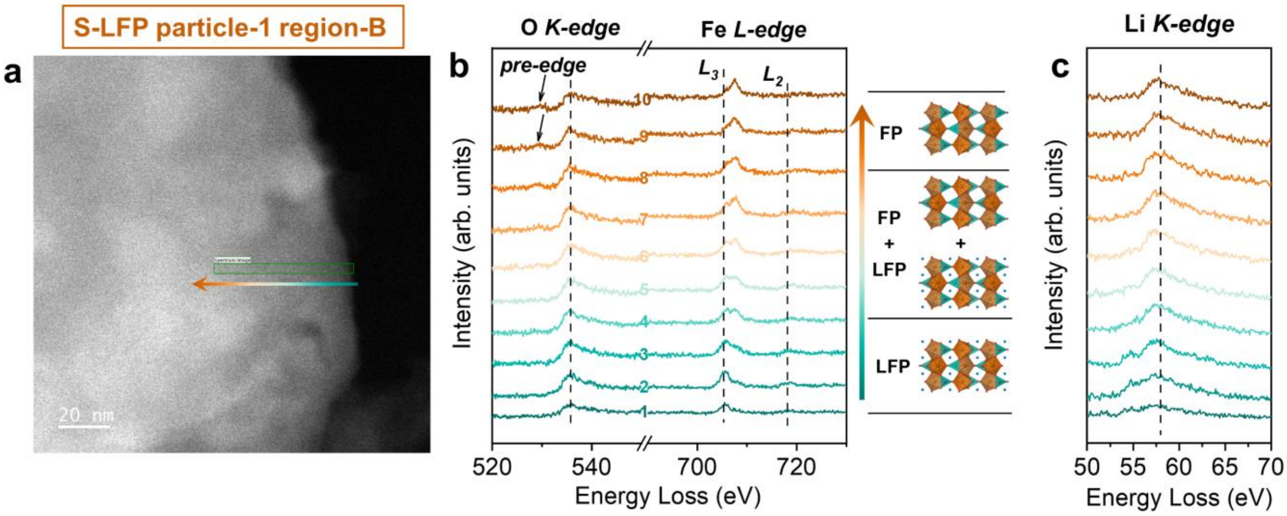

Supplementary Fig. 23 a STEM image, **b** O K-edge and Fe L-edge, and **c** Li K-edge EELS of S-LFP particle-1.

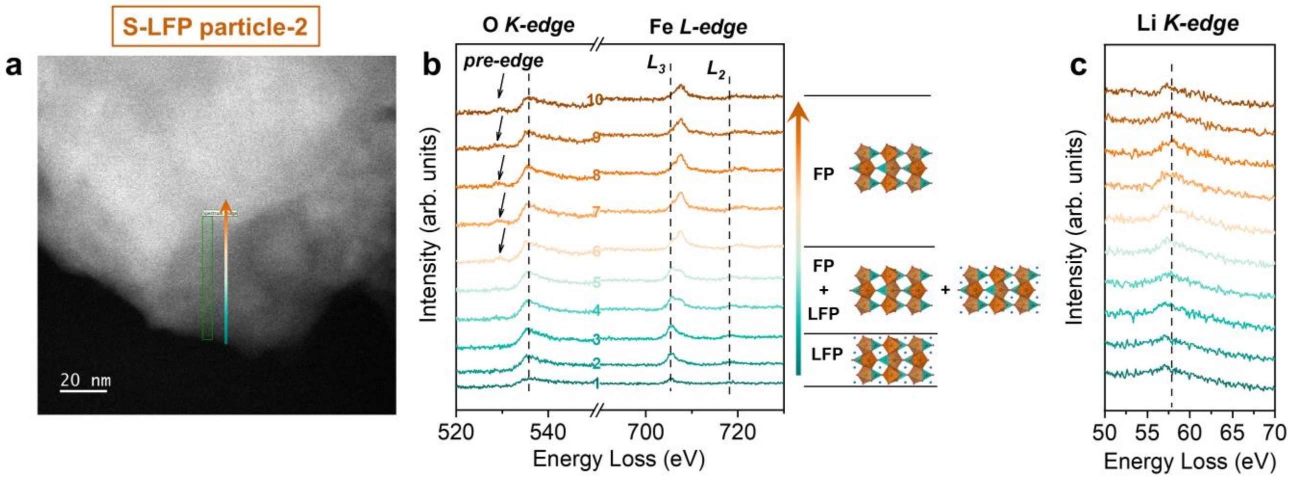

Supplementary Fig. 24 **a** STEM image, **b** O K-edge and Fe L-edge, and **c** Li K-edge EELS of S-LFP particle-2.

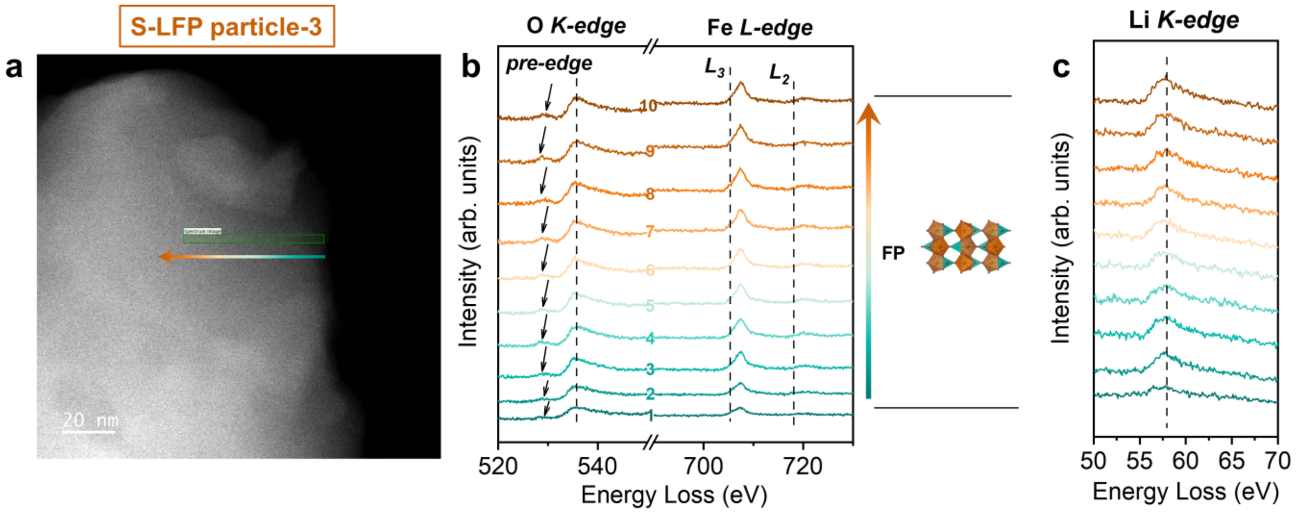

Supplementary Fig. 25 **a** STEM image, **b** O K-edge and Fe L-edge, and **c** Li K-edge EELS of S-LFP particle-3.

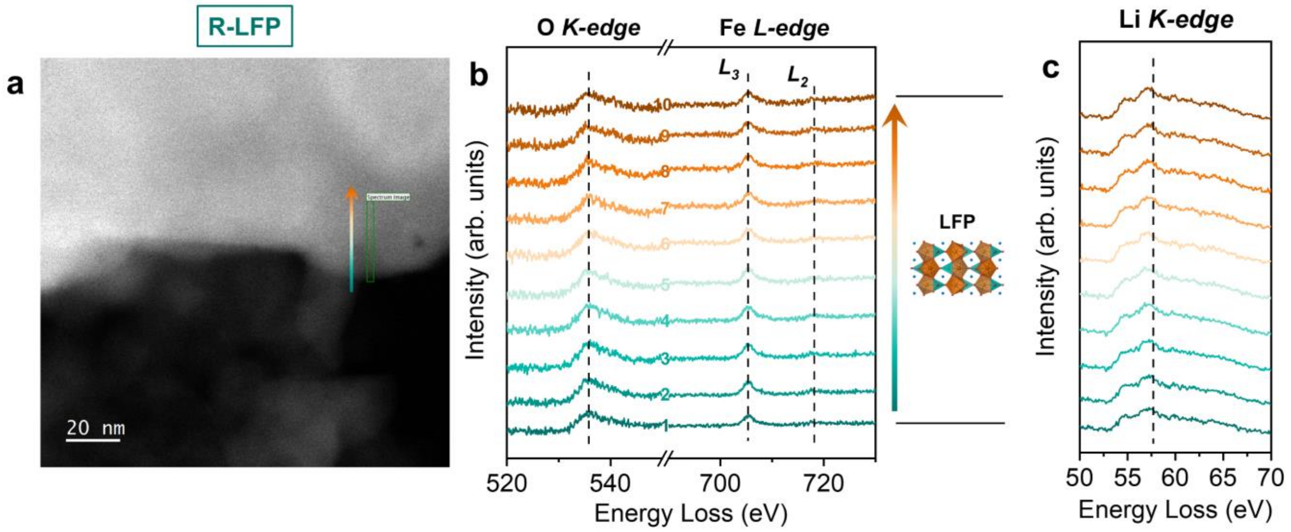

Supplementary Fig. 26 a STEM image, **b** O K-edge and Fe L-edge, and **c** Li K-edge EELS of R-LFP.

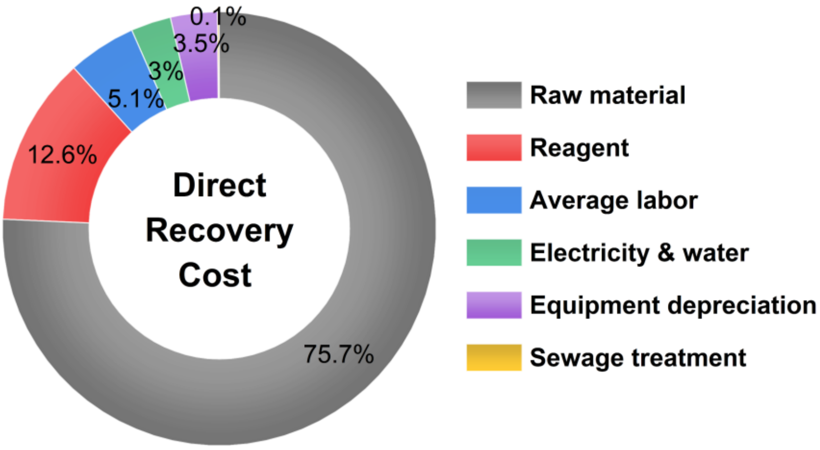

Supplementary Fig. 27 Pie chart of the % costs involved in direct recovery.

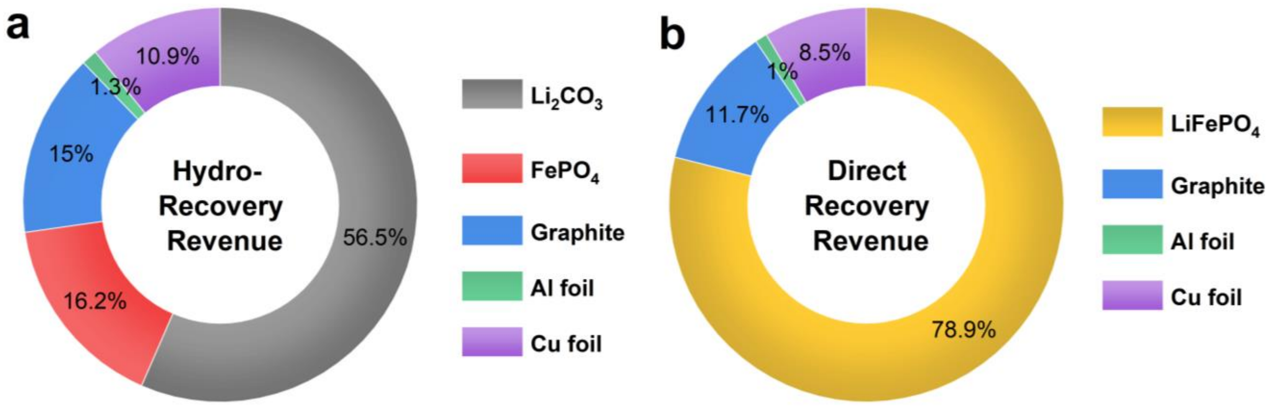

Supplementary Fig. 28 Pie charts of the % revenues obtained using **a** hydro-recovery and **b** direct recovery.

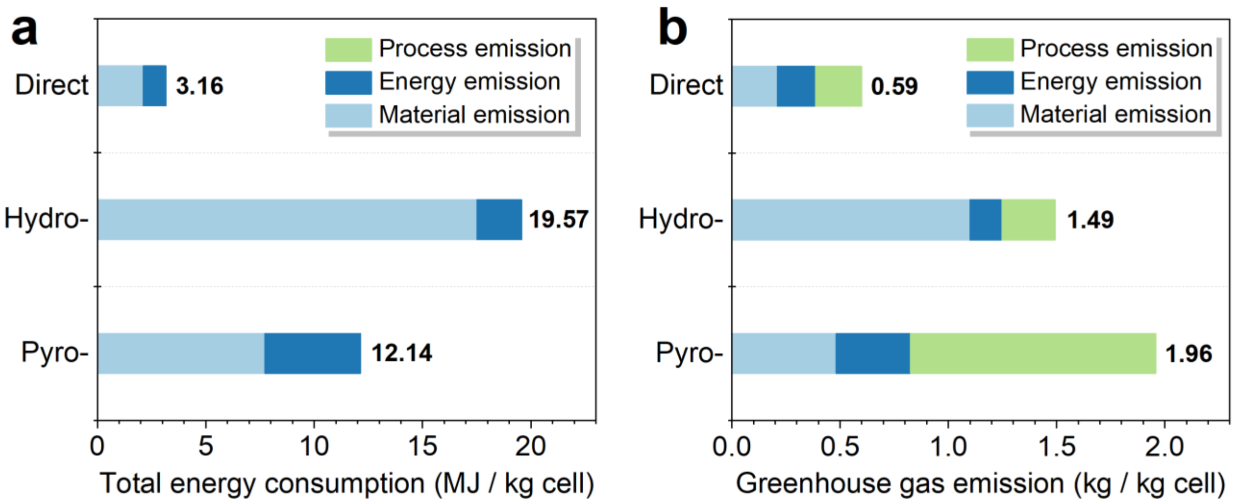

Supplementary Fig. 29 **a** Total energy consumption and **b** GHG emissions of recycling 1 kg spent LFP batteries by pyro-, hydro-, and direct recovery.

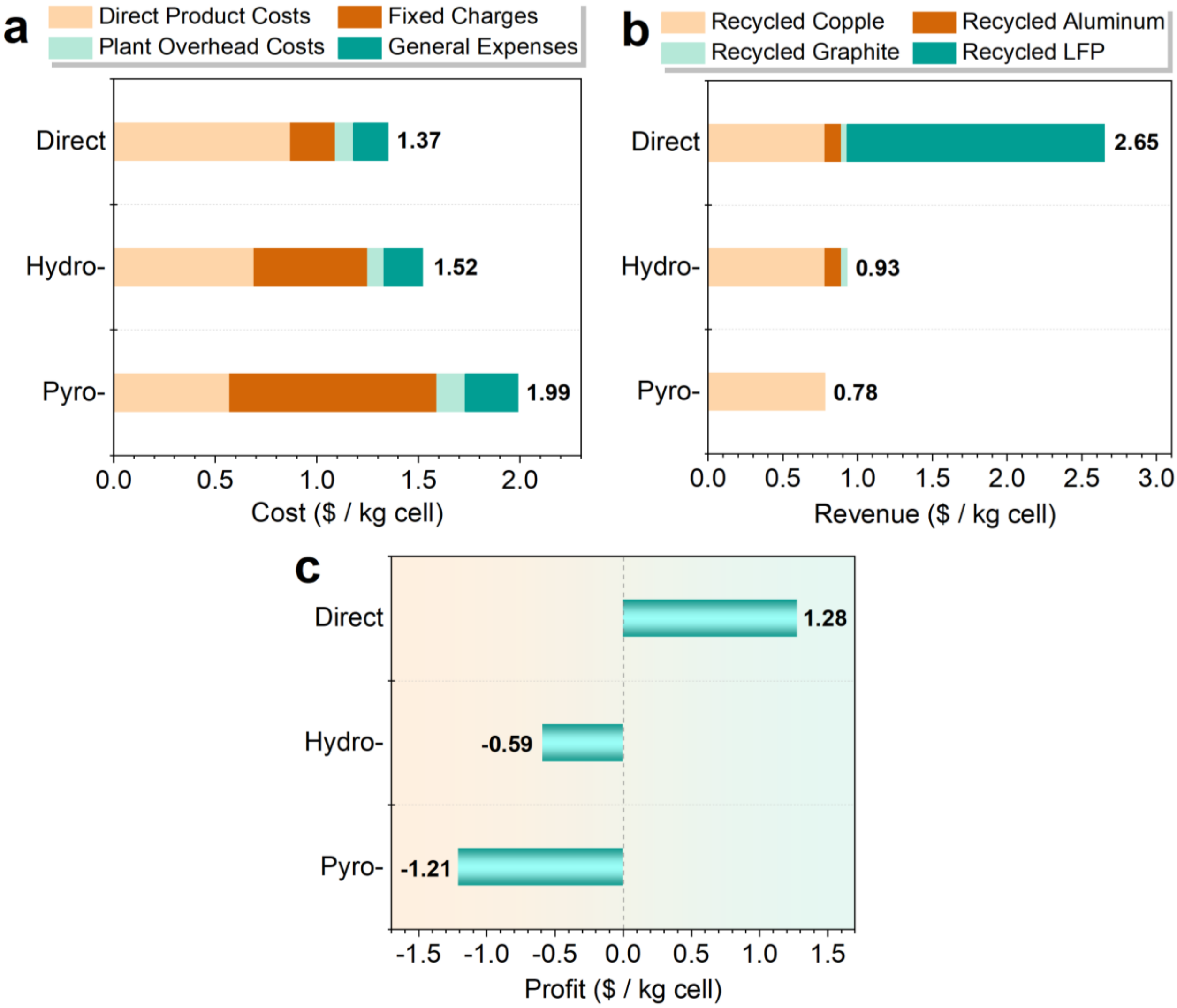

Supplementary Fig. 30 a Cost, **b** revenue, and **c** profit recycling 1 kg spent LFP batteries by pyro-, hydro-, and direct recovery.

The total costs of pyro-, hydro-, and direct recycling are $1.99, $1.52, and $1.37 per kg of cells, respectively (**Supplementary Table 10 and Supplementary Fig. 30a**). Raw materials cost is mainly responsible for the manufacturing cost in direct recycling due to the high price of lithium salt. The recycled Al, Cu, graphite, and LFP is assumed to be sold to compensate some cost of the recycling processes (**Supplementary Table 11**). Notably, the recycled LFP is mainly attributed to the total revenue for direct recycling, which is higher than pyro- and hydro- recycling (**Supplementary Fig. 30b**). Therefore, the profits of pyro-, hydro-, and direct recycling are respectively $-1.12, $-0.59, and $1.28 per kg of cells (**Supplementary Fig. 30c**).

Supplementary Table 1 The degraded LIBs used in the recovery process and the corresponding basic parameters.

	LFP	NCM	LCO
Type	Cyclinder cell	Pouch cell	Pouch cell
Manufacturer	LG (IFR18650)	Made in a laboratory	Sunwoda
Size	18*65 mm	80*55*3 mm	95*45*4 mm
Weight	~40 g	~32 g	~43 g
Voltage	3.2 V	3.7 V	3.83 V
Capacity	1.2 Ah	1.7 Ah	3.11 Ah
Profile Display	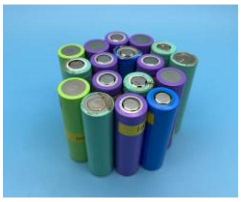	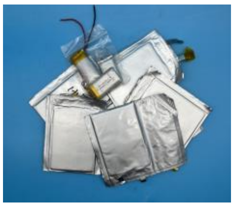	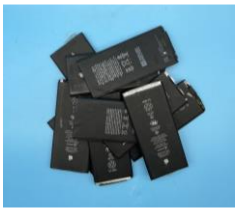

Supplementary Table 2 Element content and molar ratio of C-LFP, R-LFP and S-LFP samples based on ICP-OES results. (Note: C-LFP represents a commercial $LiFePO_4$ cathode)

	Element content (wt%)			Molar ratio	
	**Li**	**P**	**Fe**	**Li/P**	**Li/Fe**
C-LFP	4.44	18.48	33.27	1.07	1.07
R-LFP-Li_2DHBN	4.14	18.2	30.98	1.01	1.07
R-LFP-Li_2CO_3	4.41	18.11	32.36	1.08	1.09
R-LFP-LiOH	4.46	18.41	32.14	1.08	1.11
S-LFP	3.84	18.5	32.26	0.92	0.98

Supplementary Table 3 Summary of direct regeneration methods and their performance of LFP cathodes. (With annealing: No. 1-10; without annealing: No. 11-16; this work: No. 17)

**No.**	**Methods**	**Annealing conditions**	**Residual capacity (mAh/g)**	**Restored capacity (mAh/g)**	**Restored rate performance(mAh/g)**	**Ref.**
1	Solid state sintering	650 °C for 1 h	140 at 0.2C, 125 at 1C	147 at 0.2C, 130 at 1C	100 at 5C, 80 at 10C	*4*
2	Solid state sintering	700 °C for 3 h	100 at 0.2C, 115 at 1C	151 at 0.2C, 130 at 1C	120 at 10C	*5*
3	Solid state sintering	650 °C for 1 h	*Not provided.*	135 at 0.2C, 130 at 1C	108 at 5C	*6*
4	Solid state sintering	700 °C for 8 h	*Not provided.*	145 at 0.1C, 130 at 1C	105 at 5C	*7*
5	Hydrothermal	200 °C for 6 h	*Not provided.*	136 at 0.1C, 105 at 1C	84 at 2C	*8*
6	Hydrothermal	700 °C for 6 h	125 at 0.1C, 105 at 1C	166 at 0.1C, 146 at 1C	130 at 5C	*9*
7	Hydrothermal	700 °C for 5 h	101 at 1C	139 at 1C	108 at 5C, 100 at 10C	*10*
8	Solution relithiation	600 °C for 2 h	120 at 0.1C, 97 at 1C	165 at 0.1C, 150 at 1C	120 at 5C, 100 at 10C	*11*
9	Solution relithiation	600 °C for 2 h	*Not provided.*	166 at 0.1C, 150 at 1C	135 at 5C	*12*
10	Molten Salt	650 °C for 6 h	120 at 1C	130 at 1C	110 at 5C	*13*
11	Graphite prelithiation	\	65 at 0.1C	126 at 0.1C	110 at 0.5C (full cells)	*14*
12	Chemical relithiation	\	160 at 0.5C	160 at 0.5C	110 at 5C, 95 at 10C	*15*
13	Separator prelithiation	\	110 at 0.1C	160 at 0.1C, 150 at 1C	140 at 2C (full cells)	*16*
14	One-step hydrothermal	\	125 at 0.1C, 100 at 1C	146 at 0.2C, 141 at 1C	128 at 5C	*17*
15	Electrochemical re-lithiation	\	125 at 0.2C	137 at 0.2C, 134 at 1C	108 at 5C	*18*
16	Electrically driven process	\	*Not provided.*	147 at 0.1C, 134 at 1C	103 at 5C, 70 at 10C	*19*
17	Using organic lithium salt	800 °C for 6 h	102 at 0.1C, 98 at 1C	157 at 0.1C, 140 at 1C	111 at 5C, 97 at 10C	This work

Supplementary Table 4 Fitting result of equivalent circuit model for impedance parameters at different state of S-LFP sample. (OCV-open circuit voltage; C-charge; D-discharge)

SOC/DOD	R_o (Ω)	R_{ct} (Ω)	σ (Ω cm^2 $s^{-1/2}$)
OCV	1.167	47.78	33.43
C-2.5V	1.222	57.18	29.52
C-3.4V	1.207	52.67	42.42
C-3.4V	1.223	44.01	36.79
C-3.5V	1.126	38.52	45.26
C-3.6V	1.216	35.7	29.68
C-4.0V	1.23	32.92	18.67
C-4.3V	1.214	33.99	19.80
D-4.0V	1.205	36.48	21.35
D-3.4V	1.221	35.71	34.77
D-3.3V	1.232	44.05	42.25
D-3.2V	1.237	44.32	41.26
D-3.0V	1.25	43.01	45.18
D-2.5V	1.245	43.6	41.73

Supplementary Table 5 Fitting result of equivalent circuit model for impedance parameters at different state of R-LFP-Li_2DHBN sample. (OCV-open circuit voltage; C-charge; D-discharge)

SOC/DOD	R_o (Ω)	R_{ct} (Ω)	σ (Ω cm^2 s$^{-1/2}$)
OCV	4.697	62.33	60.54
C-2.5V	4.363	72.34	63.63
C-3.4V	4.315	70.16	62.30
C-3.4V	4.215	60.28	58.66
C-3.5V	4.375	22.2	45.08
C-3.6V	4.234	21.49	66.73
C-4.0V	4.205	20.36	26.95
C-4.3V	4.203	20.11	27.69
D-4.0V	4.221	21.05	27.40
D-3.4V	4.208	20.53	12.64
D-3.3V	4.309	27.07	71.92
D-3.2V	4.325	27.61	70.38
D-3.0V	4.328	28.07	73.22
D-2.5V	4.287	27.81	82.19

Supplementary Table 6 Structural parameters obtained from Rietveld refinement of the X-ray diffraction pattern of S-LFP. **Phase 1 $LiFePO_4$**: Space group: *Pnma*, a = 10.32823 Å, b = 6.00687 Å, c = 4.69310 Å, V = 291.161 Å^3, $\alpha = \beta = \gamma = 90°$, Fraction: 78.70%. **Phase 2 $FePO_4$**: Space group: *Pnma*, a = 5.79049 Å, b = 9.81996 Å, c = 4.78393 Å, V = 272.025 Å^3, $\alpha = \beta = \gamma = 90°$, Fraction: 21.30%.

		**78.70% $LiFePO_4$**					**21.30% $FePO_4$**			
**Atoms**	**Site**	**Wyckoff positions**			**Occupancy**	**Site**	**Wyckoff positions**			**Occupancy**
Li1	4a	0	0	0	0.976	NA				
Fe2	4a	0	0	0	0.024	4a	0	0	0	0.002
Fe1	4c	0.28253	0.25000	0.97203	0.976	4c	0.25000	0.27273	0.94955	0.998
Li2	4c	0.28253	0.25000	0.97203	0.024	NA				
P1	4c	0.09772	0.25000	0.42512	1	4c	0.25000	0.09681	0.40337	1
O1	4c	0.08747	0.25000	0.74366	1	4c	0.25000	0.11319	0.69996	1
O2	4c	0.45553	0.25000	0.22315	1	4c	0.25000	0.44344	0.17560	1
O3	8d	0.16361	0.04937	0.27832	1	8	0.04176	0.16562	0.25963	1

Supplementary Table 7 Structural parameters obtained from Rietveld refinement of the X-ray diffraction pattern of R-LFP-Li_2DHBN. **Phase $LiFePO_4$**: Space group: *Pnma*, a = 10.32347 Å, b = 6.00249 Å, c = 4.69709 Å, V = 291.063 Å^3, $\alpha = \beta = \gamma$ = 90°, Fraction: 100(± 0.77)%.

Atoms	Site	Wyckoff positions			Occupancy
Li1	4a	0	0	0	0.988
Fe2	4a	0	0	0	0.012
Fe1	4c	0.28291	0.25000	0.97378	0.988
Li2	4c	0.28291	0.25000	0.97378	0.012
P1	4c	0.09857	0.25000	0.42966	1
O1	4c	0.08372	0.25000	0.73929	1
O2	4c	0.44779	0.25000	0.25914	1
O3	8d	0.15309	0.04042	0.26696	1

Supplementary Table 8 Materials requirements (kg) to recycle 1 kg of spent batteries through different recycling technologies. (NR = Not Required)

	Pyro-	Hydro-	Direct
Ammonium Hydroxide	NR	0.031	NR
Hydrochloric Acid	0.21	0.012	NR
Hydrogen Peroxide	0.06	0.366	NR
Sodium Hydroxide	NR	0.561	NR
Limestone	0.30	NR	NR
Sand	0.15	NR	NR
Sulfuric Acid	NR	1.08	NR
Soda Ash	NR	0.02	NR
Lithium Hydroxide	NR	NR	0.047
Citric Acid	NR	NR	NR
Lithium Carbonate	NR	NR	0.030
Li_2DHBN	NR	NR	0.093

Supplementary Table 9 Life-cycle environmental impacts of different recycling methods.

	**Pyro-**	**Hydro-**	**Direct-**
**Total energy use in MJ per kg cell recycled**			
Total Energy	12.140	19.570	3.165
Fossil fuels	11.098	18.075	2.638
Coal	3.521	3.106	1.055
Natural gas	6.662	13.406	0.528
Petroleum	0.915	1.564	1.483
**Total Emissions in g per kg cell recycled**			
VOC	0.129	0.211	0.059
CO	0.448	0.751	0.278
NO_x	0.955	1.769	0.518
PM10	0.100	0.146	0.078
PM2.5	0.059	0.104	0.052
SO_x	1.032	22.855	0.414
BC	0.017	0.023	0.014
OC	0.014	0.033	0.017
CH_4	1.353	2.116	0.453
N_2O	0.015	0.023	0.006
CO_2	1,914	1,421	601
CO_2 (w/ C in VOC & CO)	1,915	1,423	598
GHGs	1,960	1,493	590

Supplementary Table 10 Manufacturing cost details for different recycling processes per year (10,000 tons of spent batteries).

	**Pyro-**	**Hydro-**	**Direct-**
**I. Manufacturing Cost, $/year**	$17,297,288	$13,304,279	$11,908,909
A. Direct Product Costs	$5,711,445	$6,855,798	$8,751,139
Raw Materials	$1,800,897	$4,564,966	$5,900,000
Operating labor	$323,657	$290,743	$257,829
Direct supervisory and clerical labor	$48,549	$43,611	$38,674
Utilities	$492,828	$225,111	$588,017
Maintenance and Repairs	$2,446,996	$1,347,801	$1,568,595
Operating supplies	$367,049	$202,170	$235,289
Laboratory charges	$32,366	$29,074	$25,783
Patents and royalties	$199,103	$152,321	$136,951
B. Fixed Charges	$10,176,242	$5,607,403	$2,225,221
Depreciation	$4,784,468	$2,633,067	$168,938
Local taxes	$1,957,597	$1,078,241	$954,876
Insurance	$489,399	$269,560	$313,719
Rent	$225,893	$128,979	$144,804
Financing (interest)	$2,718,885	$1,497,557	$642,884
C. Plant Overhead Costs	$1,409,601	$841,078	$932,549
**II. General Expenses, $/year**	$2,613,013	$1,927,858	$1,786,230
A. Administrative costs	$422,880	$252,323	$279,765
B. Distribution and selling costs	$1,194,618	$913,928	$821,708
C. R&D costs	$995,515	$761,607	$684,757
**III. Total Product Cost, $/year**	$19,910,301	$15,232,137	$13,695,139

Supplementary Table 11 Value of recycled materials ($/kg).

	**Pyro-**	**Hydro-**	**Direct-**
Cu	$0.78	$0.78	$0.78
Al	NA	$0.11	$0.11
Graphite	NA	$0.04	$0.04
$LiFePO_4$	NA	NA	$4.82

Original data of techno-economic analysis

Supplementary Table 12 Basic data.

The price of materials					
**No.**	**Item**	**Maket price**	**Unit**	**Update Date**	**Data Sources**
1	Degraded LFP batteries	21700.00	¥ t^{-1}	16-May-22	SMM
2	Degraded LFP powder	65000.00	¥ t^{-1}	17-May-22	SMM
3	LiOH	475500.00	¥ t^{-1}	17-May-22	SMM
4	Li_2CO_3	461500.00	¥ t^{-1}	18-May-22	SMM
5	Na_2CO_3	3000.00	¥ t^{-1}	19-May-22	100PPI
6	H_2SO_4	2500.00	¥ t^{-1}	20-May-22	100PPI
7	H_2O_2	4200.00	¥ t^{-1}	21-May-22	100PPI
8	Ar	2500.00	¥ t^{-1}	23-May-22	100PPI
9	Electricity	0.72	¥ $Kw{\cdot}h^{-1}$	21-May-22	BDB
10	Water	3.20	¥ t^{-1}	22-May-22	BDB
11	Average labor cost	73000.00	¥ a^{-1}	28-May-22	51wctt
12	sewage treatment	4.00	¥ t^{-1}	29-May-22	51wctt
13	$LiFePO_4$ cathode material	155500.00	¥ t^{-1}	30-May-22	SMM

① 1 $ = 6.698 ￥ (Update time: 2022/7/5). ② Data Sources: SMM (https://www.smm.cn/), 100PPI (http://www.100ppi.com/ppi/), BDB (http://sz.bendibao.com/), 51wctt (https://mp.weixin.qq.com/s/AoXhIwQzLquIL_Csrkw7lw).

Physical parameters			
**No.**	**Item**	**MW**	**Unit**
1	$LiFePO_4$	157.76	g mol^{-1}
2	$FePO_4$	150.82	g mol^{-2}
3	LiOH	23.95	g mol^{-1}
4	Li_2CO_3	73.89	g mol^{-1}
5	Li_2DHBN	146.88	g mol^{-1}
7	H_2SO_4	98.08	g mol^{-1}
8	H_2O_2	34.01	g mol^{-1}
9	Na_2CO_3	105.99	g mol^{-2}
10	Li	6.94	g mol^{-1}
11	Fe	55.85	g mol^{-1}
12	P	30.97	g mol^{-1}
13	O	16.00	g mol^{-1}
14	C	12.01	g mol^{-1}
15	H	1.00	g mol^{-1}
16	N	14.00	g mol^{-2}
17	Na	22.99	g mol^{-1}

Compenent in a LFP battery			
**No.**	**Item**	**Proportion**	**Unit**
1	Cathode active material	25.00	%
2	Anode active material	13.00	%
3	Al foil	6.00	%
4	Cu foil	10.00	%
5	Separator	3.00	%
6	Electrolyte	16.00	%
7	Shell	27.00	%
Ref: https://doi.org/10.1016/j.est.2020.102217			

Supplementary Table 13 Hydro- recovery cost analysis.

Cost analysis based on Hydro- recovery							
**Main**	**No.**	**Item**	**Unit Price (¥ t^{-1})**	**Dose (t)**	**Note**	**Data Source**	**Cost (¥)**
Raw material	1	Degraded battery	21700.00	1.0000	**250 kg of the degraded LFP can be sorted from per 1 ton of degraded battery.**	https://doi.org/10.1016/j.est.2020.102217	21700.00
	2	Degraded LFP		0.0000			0.00
	subtotal (¥)						21700.00
**Main**	**No.**	**Item**	**Unit Price (¥ t^{-1})**	**Dose (t)**	**Note**	**Data Source**	**Cost (¥)**
Reagent	3	H_2SO_4	2500.00	0.0886	**Used as the leaching agent to extraction lithium.**	DOI: 10.1021/acssuschemeng.7b01594	221.48
	4	H_2O_2	4200.00	0.1116	**Used as the oxidization to extraction lithium.**		468.56
	5	Na_2CO_3	3000.00	0.0541	**Used to precipitate lithium.**		162.23
	subtotal (¥)						852.28
**Main**	**No.**	**Item**	**Unit Price (¥ a^{-1})**	**Dose (a)**	**Note**	**Data Source**	**Cost (¥)**
Average labor	6	Degraded battery disassembly	73000.00	0.01		https://doi.org/10.1016/j.xcrp.2022.100741	730.00
	7	Material preparation	73000.00	0.02			1460.00
	subtotal (¥)						2190.00
**Main**	**No.**	**Item**	**Unit Price(¥ t^{-1} & ¥ kwh^{-1})**	**Dose (t & Kwh)**	**Note**	**Data Source**	**Cost (¥)**
Electricity & water	8	Water	3.20	500.00		https://doi.org/10.1016/j.xcrp.2022.100741	1600.00
	9	Electricity	0.72	500.00			360.00
	subtotal (¥)						1960.00
**Main**	**No.**	**Item**	**Unit Price (¥ t^{-1})**	**Dose (t)**	**Note**	**Data Source**	**Cost (¥)**
Equipment depreciation	10	Equipment depreciation	5000.00	0.50		DOI:10.3969/j.issn.1009-847X.2018.10.006	2500.00
	subtotal (¥)						2500.00
**Main**	**No.**	**Item**	**Unit Price (¥ t^{-1})**	**Dose (t)**	**Note**	**Data Source**	**Cost (¥)**
Sewage treatment	11	Sewage treatment	40.00	20.00		DOI:10.3969/j.issn.1009-847X.2018.10.006	800.00
	subtotal (¥)						800.00
**Total Cost (¥)**							**30002.28**

① In Supplementary Table 13, we calculated the cost per one ton of recycled cathode materials. The recycle process can be divided into three steps: disassembly of degraded battery, seperation of cathode active material, extraction of lithium.

② We assume that the failure degree of spent LFP batterry is consistent ($Li_{0.8}FePO_4$) based on Hydro- and direct recovery.

Supplementary Table 14 Direct recovery cost analysis.

Cost analysis based on Direct recovery							
**Main**	**No.**	**Item**	**Unit Price (¥ t^{-1})**	**Dose (t)**	**Note**	**Data Source**	**Cost (¥)**
Raw material	1	Degraded battery	21700.00	1.0000	**250 kg of the degraded LFP can be sorted from per 1 ton of degraded battery.**	https://doi.org/10.1016/j.est.2020.102217	21700.00
	2	Degraded LFP	0.00	0.0000			0.00
	subtotal (¥)						21700.00
**Main**	**No.**	**Item**	**Unit Price (¥ t^{-1})**	**Dose (t)**	**Note**	**Data Source**	**Cost (¥)**
Reagent	3	Li_2CO_3	461500.00	0.0117	**Used as the lithium salt to restore the degraded LFP.**	SMM	5403.82
	4	LiOH	475500.00	0.0076	**Used as the lithium salt to restore the degraded LFP.**		3609.35
	5	Li_2DHBN	199780.00	0.0233	**Used as the lithium salt to restore the degraded LFP.**		4650.05
	6	Ar	2500.00	0.01	**Used as the protective atmospherer during regeneraiton process.**		25.00
	subtotal (¥)						4650.05
**Main**	**No.**	**Item**	**Unit Price (¥ a^{-1})**	**Dose (a)**	**Note**	**Data Source**	**Cost (¥)**
Average labor	7	Disassemble degraded battery	73000.00	0.01		https://doi.org/10.1016/j.xcrp.2022.100741	730.00
	8	Material preparation	73000.00	0.01			730.00
	subtotal (¥)						1460.00
**Main**	**No.**	**Item**	**Unit Price(¥ t^{-1} & ¥ kwh^{-1})**	**Dose (t & Kwh)**	**Note**	**Data Source**	**Cost (¥)**
Electricity & water	9	Water	3.20	200.00		https://doi.org/10.1016/j.xcrp.2022.100741	640.00
	10	Electricity	0.72	300.00			216.00
	subtotal (¥)						856.00
**Main**	**No.**	**Item**	**Unit Price (¥ t^{-1})**	**Dose (t)**	**Note**	**Data Source**	**Cost (¥)**
Equipment depreciation	11	Equipment depreciation	5000.00	0.20		DOI:10.3969/j.issn.1009-847X.2018.10.006	1000.00
	subtotal (¥)						1000.00
**Main**	**No.**	**Item**	**Unit Price (¥ t^{-1})**	**Dose (t)**	**Note**	**Data Source**	**Cost (¥)**
Sewage treatment	12	Sewage treatment	40.00	1.00		DOI:10.3969/j.issn.1009-847X.2018.10.006	40.00
	subtotal (¥)						40.00
**Total Cost (¥)**							**29706.05**

① In Supplementary Table 14, we calculated the cost per one ton of recycled cathode materials. The recycle process can be divided into three steps: disassembly of degraded battery, seperation of cathode active material, regeneration of LFP material.
② We assume that the failure degree of spent LFP batterry is consistent ($Li_{0.8}FePO_4$) based on Hydro- and direct recovery.

Different Li content in degraded LFP (Li_xFePO_4)			
0.80		Cost (￥)	Total Cost (￥)
**The consumption of Li_2CO_3 (t)**	0.0117	5403.82	30459.82
**The consumption of LiOH (t)**	0.0076	3609.35	28665.35
**The consumption of Li_2DHBN (t)**	0.0233	4650.05	29706.05
Note: We assume that different residual Li content determines the consumption of lithium salt in regeneration process.			

Cost analysis of 1 ton Li_2DHBN				
**Raw materials**	**Unit Price (￥ t^{-1})**	**Dose (t)**	**Data Source**	**Cost (￥)**
3,4-dihydroxybenzonitrile	100000.00	0.919	https://b2b.baidu.com/	91900.00
tetrahydrofuran (THF)	8000.00	12.000		96000.00
lithium hydride (LiH)	110000.00	0.108		11880.00
subtotal (￥)	199780.00			

Supplementary Table 15 Hydro- recovery revenue analysis.

Revenue analysis based on Hydro- recovery								
**No.**	**Item**	**Material**	**Recovery yield (%)**	**Production (t)**	**Market price (￥ t^{-1})**	**Revenue (￥)**	**Update Date**	**Data Sources**
1	**Lithium salt**	Li_2CO_3	95	0.0445	461,500.00	20534.50	16-May-22	SMM
2	**Precursor**	$FePO_4$	95	0.2271	26,000.00	5903.36	16-May-22	SMM
3	**Anode material**	Graphite	98	0.1274	42,900.00	5465.46	16-May-22	SMM
4	**Curret collector**	Al foil	90	0.0540	8,671.00	468.23	16-May-22	https://doi.org/10.1016/j.joule.2020.10.008
5	**Curret collector**	Cu foil	90	0.0900	44,022.00	3961.98	16-May-22	https://doi.org/10.1016/j.joule.2020.10.009
6	**Separator**		98	0.0294	NA	NA		
7	**Electrolyte**		90	0.1440	NA	NA		
8	**Shell**		98	0.1568	NA	NA		
subtotal (￥) 32314.9741								
Note: ① We assume that the failure degree of spent LFP batterry is consistent ($Li_{0.8}FePO_4$) based on Hydro- and direct recovery. ② The proportion of each compenent is 27% of cathode material, 13% of graphite, 6% of Al foil, 10% of Cu foil, 3% of separator, 16% of electrolyte, and 27% of shell (Ref: https://doi.org/10.1016/j.est.2020.102217). ③ The value of Li_2CO_3, $FePO_4$, and graphite is calculated based on the real market price from SMM. ④ The value of separator, electrolyte and shell is hard to assess in real process, therefore, these components are not included in our revenue analysis.								

**Different Li content in degraded LFP (Li_xFePO_4)**		
0.80		**Revenue (￥)**
**The production of Li_2CO_3 (t)**	0.0445	20534.50
Note: We assume that different residual Li content determines the production of lithium salt in Hydro- recovery process.		

Supplementary Table 16 Direct recovery revenue analysis.

Revenue analysis based on Direct recovery								
**No.**	**Item**	**Material**	**Recovery yield (%)**	**Production (t)**	**Market price (￥ t^{-1})**	**Revenue (￥)**	**Update Date**	**Data Sources**
1	**Cathode Material**	$LiFePO_4$	95	0.2375	155,500.00	36931.25	16-May-22	SMM
2	**Anode material**	Graphite	98	0.1274	42,900.00	5465.46	16-May-22	SMM
3	**Curret collector**	Al foil	90	0.0540	8,671.00	468.23	16-May-22	https://doi.org/10.1016/j.joule.2020.10.009
4	**Curret collector**	Cu foil	90	0.0900	44,022.00	3961.98	16-May-22	https://doi.org/10.1016/j.joule.2020.10.009
5	**Separator**		95	0.0285	NA	NA		
6	**Electrolyte**		90	0.1440	NA	NA		
7	**Shell**		98	0.2646	NA	NA		
subtotal (￥) 41213.3740								
Note: ① We assume that the failure degree of spent LFP batterry is consistent ($Li_{0.8}FePO_4$) based on Hydro- and direct recovery. ② The proportion of each compenent is 27% of cathode material, 13% of graphite, 6% of Al foil, 10% of Cu foil, 3% of separator, 16% of electrolyte, and 27% of shell (Ref: https://doi.org/10.1016/j.est.2020.102217). ③ The value of regenerated $LiFePO_4$ is calculated based on the real market price from SMM. ④The value of separator, electrolyte and shell is hard to assess in real process, therefore, these compenents are not included in our revenue analysis.								

Supplementary Table 17 Hydro- recovery cost results.

Hydro- & Direct recovery cost results															
No.	Li Content	Hydro- recovery cost（¥）							Direct recovery cost（¥）						
		Raw material	Reagent	Average labor	Electricity & water	Equipment depreciation	Sewage treatment	Total cost	Raw material	Reagent	Average labor	Electricity & water	Equipment depreciation	Sewage treatment	Total cost
1	0.80	21700.00	852.28	2190.00	1960.00	2500.00	800.00	30002.28	21700.00	4650.05	1460.00	856.00	1000.00	40.00	29706.05
2	0.60	21700.00	852.28	2190.00	1960.00	2500.00	800.00	30002.28	21700.00	9300.10	1460.00	856.00	1000.00	40.00	34356.10
3	0.40	21700.00	852.28	2190.00	1960.00	2500.00	800.00	30002.28	21700.00	13950.16	1460.00	856.00	1000.00	40.00	39006.16
4	0.20	21700.00	852.28	2190.00	1960.00	2500.00	800.00	30002.28	21700.00	18600.21	1460.00	856.00	1000.00	40.00	43656.21

Supplementary Table 18 Direct recovery cost results.

Hydro- & Direct recovery revenue results												
No.	Li Content	Hydro- recovery revenue (¥)						Direct recovery revenue（¥）				
		Li_2CO_3	$FePO_4$	Graphite	Al foil	Cu foil	Total revenue	$LiFePO_4$	Graphite	Al foil	Cu foil	Total revenue
1	0.80	20534.50	5903.36	5465.46	468.23	3961.98	36333.53	36931.25	5465.46	468.23	3961.98	46826.92
2	0.60	15400.87	5903.36	5465.46	468.23	3961.98	31199.90	36931.25	5465.46	468.23	3961.98	46826.92
3	0.40	10267.25	5903.36	5465.46	468.23	3961.98	26066.28	36931.25	5465.46	468.23	3961.98	46826.92
4	0.20	5133.62	5903.36	5465.46	468.23	3961.98	20932.65	36931.25	5465.46	468.23	3961.98	46826.92

Supplementary Table 19 Profit calculation.

Recovery profit results			
**No.**	**Li Content**	**Hydro- profit (¥)**	**Direct profit (¥)**
1	0.80	6331.25	17120.87
2	0.60	1197.62	12470.82
3	0.40	-3936.00	7820.76
4	0.20	-9069.63	3170.71

Supplementary References

1. Hong, L., *et al.* Two-dimensional lithium diffusion behavior and probable hybrid phase transformation kinetics in olivine lithium iron phosphate. *Nat. Commun.* **8**, 1194 (2017).

2. Gu, W., *et al.* Recyclable, self-healing solid polymer electrolytes by soy protein-based dynamic network. *Adv. Sci.* **9**, 2103623 (2022).

3. Shen, Z., Cao, L., Rahn, C. D. & Wang, C. Y. Least squares galvanostatic intermittent titration technique (LS-GITT) for accurate solid phase diffusivity measurement. *J. Electrochem. Soc.* **160**, A1842-A1846 (2013).

4. Li, X. L., Zhang, J., Song, D. W., Song, J. S. & Zhang, L. Q. Direct regeneration of recycled cathode material mixture from scrapped $LiFePO_4$ batteries. *J. Power Sources* **345**, 78-84 (2017).

5. Li, J., Wang, Y., Wang, L. H., Liu, B. & Zhou, H. M. A facile recycling and regeneration process for spent $LiFePO_4$ batteries. *J. Mater. Sci.: Mater. Electron.* **30**, 14580-14588 (2019).

6. Chen, J. P., Li, Q. W., Song, J. S., Song, D. W., Zhang, L. Q. & Shi, X. X. Environmentally friendly recycling and effective repairing of cathode powders from spent $LiFePO_4$ batteries. *Green Chem.* **18**, 2500-2506 (2016).

7. Song, X., *et al.* Direct regeneration of cathode materials from spent lithium iron phosphate batteries using a solid phase sintering method. *RSC Adv.* **7**, 4783-4790 (2017).

8. Song, Y. F., *et al.* Regeneration of $LiFePO_4$ from spent lithium-ion batteries via a facile process featuring acid leaching and hydrothermal synthesis. *Green Chem.* **23**, 3963-3971 (2021).

9. Chen, B. B., *et al.* Direct regeneration and performance of spent $LiFePO_4$ via a green efficient hydrothermal technique. *J. Alloys Compd.* **924**, 166487 (2022).

10. Jia, K., *et al.* Long-life regenerated $LiFePO_4$ from spent cathode by elevating the *d*-band center of Fe. *Adv. Mater.*, e2208034 (2022). Preprint at https://doi.org/10.1002/adma.202208034

11. Xu, P. P., *et al.* Efficient direct recycling of lithium-ion battery cathodes by targeted healing. *Joule* **4**, 2609-2626 (2020).

12. Fan, M., *et al.* Structural restoration of degraded $LiFePO_4$ cathode with enhanced kinetics using residual lithium in spent graphite anodes. *CCC Chem.*, 1-13 (2022). Preprint at https://doi.org/10.31635/ccschem.022.202201996

13. Liu, X., Wang, M. M., Deng, L. P., Cheng, Y. J., Gao, J. & Xia, Y. G. Direct regeneration of spent

lithium iron phosphate via a low-temperature molten salt process coupled with a reductive environment. *Ind. Eng. Chem. Res.* **61**, 3831-3839 (2022).

14. Wang, T., *et al.* Direct regeneration of spent $LiFePO_4$ via a graphite prelithiation strategy. *Chem. Commun.* **56**, 245-248 (2019).

15. Wu, C., *et al.* Direct regeneration of spent Li-ion battery cathodes via chemical relithiation reaction. *ACS Sustain. Chem. Eng.* **9**, 16384-16393 (2021).

16. Fan, M., *et al.* In situ electrochemical regeneration of degraded $LiFePO_4$ electrode with functionalized prelithiation separator. *Adv. Energy Mater.* **12**, 2103630 (2022).

17. Jing, Q. K., Zhang, J. L., Liu, Y. B., Zhang, W. J., Chen, Y. Q. & Wang, C. Y. Direct regeneration of spent $LiFePO_4$ cathode material by a green and efficient one-step hydrothermal method. *ACS Sustain. Chem. Eng.* **8**, 17622-17628 (2020).

18. Zhou, S. Y., *et al.* Direct recovery of scrapped $LiFePO_4$ by a green and low-cost electrochemical re-lithiation method. *Green Chem.* **24**, 6278-6286 (2022).

19. Peng, D., *et al.* Efficient regeneration of retired $LiFePO_4$ cathode by combining spontaneous and electrically driven processes. *Green Chem.* **24**, 4544-4556 (2022).
